# Charged residues next to transmembrane regions revisited: “Positive-inside rule” is complemented by the “negative inside depletion/outside enrichment rule”

**DOI:** 10.1186/s12915-017-0404-4

**Published:** 2017-07-24

**Authors:** James Alexander Baker, Wing-Cheong Wong, Birgit Eisenhaber, Jim Warwicker, Frank Eisenhaber

**Affiliations:** 1 Bioinformatics Institute, Agency for Science Technology and Research (A*STAR), 30 Biopolis Street #07-01, Matrix, Singapore, 138671 Singapore; 2 School of Chemistry, Manchester Institute of Biotechnology, 131 Princess Street, Manchester, M1 7DN UK; 3 School of Computer Engineering (SCE), Nanyang Technological University (NTU), 50 Nanyang Drive, Singapore, 637553 Singapore

**Keywords:** Amino acid distribution, Genome-wide statistical study, Membrane protein, Negative-not-inside/negative-outside rule, Protein topology prediction, Proteomics, Transmembrane helix, Transmembrane region prediction

## Abstract

**Background:**

Transmembrane helices (TMHs) frequently occur amongst protein architectures as means for proteins to attach to or embed into biological membranes. Physical constraints such as the membrane’s hydrophobicity and electrostatic potential apply uniform requirements to TMHs and their flanking regions; consequently, they are mirrored in their sequence patterns (in addition to TMHs being a span of generally hydrophobic residues) on top of variations enforced by the specific protein’s biological functions.

**Results:**

With statistics derived from a large body of protein sequences, we demonstrate that, in addition to the positive charge preference at the cytoplasmic inside (positive-inside rule), negatively charged residues preferentially occur or are even enriched at the non-cytoplasmic flank or, at least, they are suppressed at the cytoplasmic flank (negative-not-inside/negative-outside (NNI/NO) rule). As negative residues are generally rare within or near TMHs, the statistical significance is sensitive with regard to details of TMH alignment and residue frequency normalisation and also to dataset size; therefore, this trend was obscured in previous work. We observe variations amongst taxa as well as for organelles along the secretory pathway. The effect is most pronounced for TMHs from single-pass transmembrane (bitopic) proteins compared to those with multiple TMHs (polytopic proteins) and especially for the class of simple TMHs that evolved for the sole role as membrane anchors.

**Conclusions:**

The charged-residue flank bias is only one of the TMH sequence features with a role in the anchorage mechanisms, others apparently being the leucine intra-helix propensity skew towards the cytoplasmic side, tryptophan flanking as well as the cysteine and tyrosine inside preference. These observations will stimulate new prediction methods for TMHs and protein topology from a sequence as well as new engineering designs for artificial membrane proteins.

**Electronic supplementary material:**

The online version of this article (doi:10.1186/s12915-017-0404-4) contains supplementary material, which is available to authorized users.

## Background

Two decades ago, the classic concept of a transmembrane helical region was a rather simple story: Typical transmembrane proteins were thought to be anchored in the membrane by membrane-spanning bundles of non-polar α-helices of roughly 20 residues in length, with a consistent orientation of being perpendicular to the membrane surface. Although this is broadly true, hundreds of high-quality membrane structures have elucidated that membrane-embedded helices can adopt a plethora of lengths and orientations within the membrane. They are capable of just partially spanning the membrane, spanning using oblique angles, and even lying flat on the membrane surface [1, 2]. The insertion and formation of the transmembrane helices (TMHs) follow a complex thermodynamic equilibrium [3]. From the biological function point of view, many TMHs have multiple roles besides being just hydrophobic anchors; for example, certain TMHs have been identified as regulators of protein quality control and trafficking mechanisms [4]. As these additional biological functions are mirrored in the TMHs’ sequence patterns, TMHs can be classified as simple (just hydrophobic anchors) and complex sequence segments [5–7].

The relationship between sequence patterns in and in the vicinity of TMHs and their structural and functional properties, as well as their interaction with the lipid bilayer membrane, has been a field of intensive research in the last three decades [8]. Besides the span of generally hydrophobic residues in the TMH, there are other trends in the sequence such as a saddle-like distribution of polar residues (depressed incidence of charged residues in the TMH itself), an enriched occurrence of positively charged residues in the cytosolic flanking regions as well as an increased likelihood of tryptophan and tyrosine at either flank edge [9–14]. These properties vary somewhat in length and intensity between various biological organelle membranes, between prokaryotes and eukaryotes [15] and even amongst eukaryotic species studied due to slightly different membrane constraints [9, 16]. These biological dispositions are exploitable in terms of transmembrane region prediction in query protein sequences [17, 18], and tools such as the quite reliable TMHMM (software for predicting TMHs based on a hidden Markov model), Phobius or the dense alignment surface-transmembrane filter (DAS-TMfilter) represent today’s prediction limit of TMHs’ hydrophobic cores within the protein sequence [19–25]. The prediction accuracy for true positives and negatives is reported to be close to 100%, and the remaining main cause of false positive prediction is hydrophobic α-helices completely buried in the hydrophobic core of proteins. Note that reliable prediction of TMHs and protein topology is a strong restriction for protein function of even otherwise non-characterised proteins [26–28] and thus provides very valuable information.

The “positive-inside rule” reported by von Heijne [2, 12] postulates the preferential occurrence of positively charged residues (lysine and arginine) at the cytoplasmic edge of TMHs. The practical value of positively charged residue sequence clustering in topology prediction of TMHs was first shown for the plasmalemma in bacteria [12, 29]. As a trend, the positive-inside rule has since been confirmed with statistical observations for most membrane proteins and biological membrane types [13, 30–32]. However, more recent evidence suggests that, in thylakoid membranes, the positive-inside rule is less applicable due to the co-occurrence of aspartic acid and glutamic acid residues together with positively charged residues [16].

The positive-inside rule also received support from protein engineering experiments that revealed conclusive evidence for positive charges as a topological determinant [12, 33–35]. Mutational experiments demonstrated that charged residues, when inserted into the centre of the helix, had a large effect on insertion capabilities of the TMH via the translocon. Insertion becomes more unfavourable when the charge is placed closer to the TMH core [36].

It remains unclear exactly why and how the positive charge determines topology from a biophysical perspective. Positively charged residues are suggested to be stronger determinants of topology than negatively charged residues due to a dampening of the translocation potential of negatively charged residues. This dampening factor is the result of protein-lipid interactions with the net-zero-charged phospholipid phosphatidylethanolamine and other neutral lipids. This effect favours cytoplasmic retention of positively charged residues [37].

The recent accumulation of transmembrane protein sequences and structures allowed us to revisit the problem of charged residue distribution in TMHs (see also http://blanco.biomol.uci.edu/mpstruc/). For example, whilst β-sheets contain charged residues in the transmembrane region, α-helices generally do not [38]. Large-scale sequence analysis of TMHs from various organelle membrane surfaces in eukaryotic proteomes confirms the clustering of positive charge having a statistical bias for the cytosolic side of the membrane. At the same time, there are many TMH exception examples to the positive-inside rule; however, as a trend, topology can be determined by simply looking for the most positive loop region between helices [9, 13].

When the observation of positively charged residues preferentially localised at the cytoplasmic edge of TMHs emerged, it was also asked whether negatively charged residues work in concert with TMH orientation. It was shown that a single additional lysine residue can reverse the topology of a model *Escherichia coli* protein, whereas many more negatively charged residues are needed to achieve the same [35]. Nevertheless, a sufficiently large negative charge can overturn the positive-inside rule [39, 40]; thus indeed, negative residues are topologically active to a point. Negatively charged residues were observed in the flanks of TMHs [13], especially in those of marginally hydrophobic transmembrane regions [41]. It is known that the negatively charged acidic residues in transmembrane regions have a non-trivial role in the biological context. In *E. coli*, negative residues experience electrical pulling forces when travelling through the SecYEG translocon, indicating that negative charges are biologically relevant during the electrostatic interactions of insertion [42, 43].

Unfortunately, there is a problem with statistical evidence for preferential negative charge occurrence next to TMH regions. Early investigations indicated that overall both positive and negative charge were influential topology factors; this idea was dubbed the charge balance rule. If true, one would also expect to see a skew in the negative charge distribution if a cooperation between oppositely charged residues oriented a TMH [29, 44]. It might be expected that, if positive residues force the loop or tail to stay inside, negative residues would be drawn outside, and the topology would be determined, not unlike electrophoresis. Yet, there are plenty of individual protein examples but no conclusive statistical evidence in the current literature for a negatively charged skew [9, 13, 14, 16, 31, 45].

There are many observations described in the literature that charged residues determine topology more predictably in single-pass proteins than in multi-pass TMHs [40, 46]. It is thought that the charges only determine the initial orientation of the TMH in the biological membrane; yet, the ultimate orientation must be determined together with the totality of subsequent downstream regions [47].

With sequence-based hydrophobicity and volume analysis and consensus sequence studies, Sharpe et al. [9] demonstrated that there is asymmetry in the intra-membranous space of some membranes. Crucially, this asymmetry differs amongst the membranes of various organelles. They conclude that there are general differences between the lipid composition and organisation in membranes of the Golgi and endoplasmic reticulum (ER). Functional aspects are also important. For example, the abundance of serines in the region following the luminal end of Golgi TMHs appears to reflect the fact that this part of many Golgi enzymes forms a flexible linker that tethers the catalytic domain to the membrane [9].

A study by Baeza-Delgado et al. [13] analysed the distribution of amino acid residue types in TMHs in 170 integral membrane proteins from a manually maintained database of experimentally confirmed TMPs (MPtopo [48]) as well as in 930 structures from the Protein Data Bank (PDB). As expected, half of the natural amino acids are equally distributed along TMHs, whereas aromatic, polar and charged amino acids along with proline are biased near the flanks of the TMHs. Unsurprisingly, leucine and other non-polar residues are far more abundant than the charged residues in the transmembrane region [9].

In this work, we revisit the issue of statistical evidence for the preferential distribution of negatively charged (and a few other) residues within and nearby TMHs. We rely on the improved availability of comprehensive and large sequence and structure datasets for transmembrane proteins. We also show that several methodological aspects have hindered previous studies [9, 13, 16] from seeing the consistent non-trivial skew for negatively charged residues disfavouring the cytosolic interfacial region and/or preferring the outside flank. First, we show that acidic residues are especially rare within and in the close sequence environment of TMHs, even when compared to positively charged lysine and arginine. Second, therefore, the manner of normalisation is critical: Taken together with the difficulty of properly aligning TMHs relative to their boundaries, column-wise frequency calculations relative to all amino acid types as in previous studies will blur possible preferential localisations of negative charges in the sequence. However, the outcome changes when we ask where a negative charge occurs in the sequence relative to the total amount of negative charges in the respective sequence region. Thus, by accounting for the rarity of acidic residues with sensitive normalisation, the “non-negative inside rule/negative-outside rule” is clearly supported by the statistical data. We find that minor changes in the flank definitions, such as taking the TMH boundaries from the database or generating flanks by centrally aligning TMHs and applying some standardised TMH length, do not have a noticeable influence on the charge bias detected.

Third, there are significant differences in the distribution of amino acid residues between single-pass and multi-pass transmembrane regions in both the intra-membrane helix and the flanking regions, with further variations introduced by taxa and by the organelles along the secretory pathway. Importantly, we find that it is critical to weigh down the effect of TMHs in multi-pass transmembrane proteins with no or super-short flanks to observe statistical significance for the charge bias. Bluntly stated, if there are no flanks of sufficient length, there is also no negative charge bias to be observed.

The charge bias effect is even clearer when a classification of TMHs into so-called simple TMHs (which, as a trend, are mostly single-pass and mere anchors) and so-called complex ones (which typically have functions beyond anchorage) is considered [5–7]. We also observe parallel skews with regard to leucine, tyrosine, tryptophan and cysteine distributions. With these large-scale datasets and a sensitive normalisation approach, new sequence features are revealed that provide spatial insight into TMH membrane anchoring, recognition, helix-lipid, and helix-helix interactions.

## Results

### Acidic residues within and nearby TMH segments are rare

In order to reliably compare the amino acid sequence properties of TMHs, we assembled datasets of TMH proteins from what are likely to be the best in terms of quality and comprehensiveness of annotation in eukaryotic and prokaryotic representative genomes, as well as composite datasets to represent larger taxonomic groups and to consider subcellular locations (see Table 1). In total, 3292 single-pass TMH segments and 29,898 multi-pass TMH segments were extracted from various UniProt [49] text files according to TRANSMEM annotation (download dated 20-03-2016). The UniProt datasets used included only manually curated records; however, it is still necessary to check for systematic bias due to the prediction methods used by UniProt for TMH annotation in the majority of cases without direct experimental evidence. Therefore, a fully experimentally verified dataset was also generated for comparison. The representative 1544 single-pass and 15,563 TMHs were extracted from the manually curated experimentally verified TOPDB [50] database (download dated 21-03-2016) referred to as ExpAll here (Table1). TMH organelle residency is defined according to UniProt annotation. To ensure reliability, organelles were only analysed from a representative redundancy-reduced protein dataset of the most well-studied genome: *Homo sapiens* (referred to as UniHuman herein). The several datasets from UniProt are subdivided into different human organelles (UniPM, UniER, UniGolgi) and taxonomical groups (UniHuman, UniCress, UniBacilli, UniEcoli, UniArch, UniFungi) as described in Table 1 (see also the Methods section). As will be shown below, these various datasets allow us to validate our findings for a variety of conditions, namely with regard (1) to experimental verification of TMHs, (2) to origin from various species and taxonomic groups, (3) to the number of TMHs in the same protein as well as (4) to subcellular localisation. Datasets and programs used in this work can be downloaded from http://mendel.bii.a-star.edu.sg/SEQUENCES/NNI/.

**Table 1:** Acidic residues are rarer in TMHs of single-pass proteins than in TMHs of multi-pass proteins

Dataset	Acidic residues (D and E)			Aspartic acid (D only)			Glutamic acid (E only)		
	μ SP	μ MP	*H* statistic *P* value	μ SP	μ MP	*H* statistic *P* value	μ SP	μ MP	*H* statistic *P* value
*ExpAll*	0.086	0.309	148.1 4.50E-34	0.045	0.157	40.3 2.13E-10	0.042	0.161	46.6 8.64E-12
*UniHuman*	0.076	0.398	316.5 8.31E-71	0.034	0.191	91.6 1.05E-21	0.042	0.207	100.3 1.33E-23
*UniER*	0.106	0.430	34.4 4.39E-9	0.061	0.161	8.0 4.72E-3	0.045	0.268	26.8 2.24E-7
*UniGolgi*	0.097	0.381	39.8 2.88E-10	0.043	0.180	19.4 1.05E-5	0.053	0.201	20.2 7.01E-6
*UniPM*	0.039	0.400	121.0 3.86E-28	0.016	0.187	32.7 1.06E-8	0.022	0.213	36.9 1.26E-9
*UniCress*	0.062	0.434	163.5 1.99E-37	0.036	0.198	32.5 1.20E-8	0.025	0.241	66.0 4.59E-16
*UniFungi*	0.177	0.349	43.1 5.14E-11	0.044	0.166	24.5 7.60E-7	0.133	0.183	4.6 0.033
*UniBacilli*	0.089	0.352	24.1 9.16E-7	0.048	0.185	11.2 8.27E-4	0.040	0.176	12.3 4.54E-5
*UniEcoli*	0.148	0.315	2.7 0.100	0.111	0.150	0.1 0.729	0.037	0.163	2.2 0.140
*UniArch*	0.438	0.606	1.8 0.183	0.083	0.344	11.2 8.33E-4	0.354	0.247	3.5 0.0624

The statistical results when comparing the number of acidic residues in single-pass or multi-pass TMHs within their database-defined limits and excluding any flanks. The number of helices per dataset can be found in Table 2 for single-pass TMHs and Table 3 for multi-pass helices. µSP is the average number of the respective residues per helix in TMHs from single-pass proteins, while µMP is the average number of the respective residues per TMH from multi-pass proteins. The Kruskal-Wallis test scores (*H* statistics) were calculated for the numbers of aspartic acid and glutamic acid residues in each helix from single-pass and the number of aspartic acid and glutamic acid residues in each helix from multi-pass TMHs

**Fig. 1:**
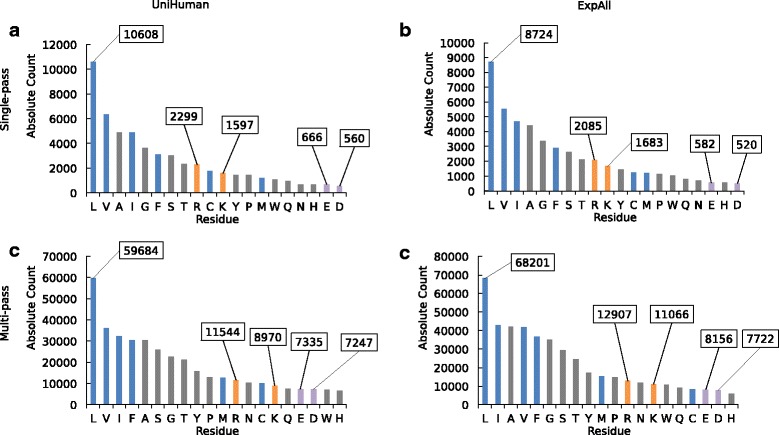
Negatively charged amino acids are amongst the rarest residues in TMHs and ±5 flanking residues. Bar charts of the abundance of each amino acid type in the TMHs with flank lengths of the accompanying ±5 residues from the (**a**) UniHuman single-pass proteins, (**b**) ExpAll single-pass proteins, (**c**) UniHuman multi-pass proteins, and (**d**) ExpAll multi-pass proteins. Amino acid types on the *horizontal axis* are listed in descending count. The bars were coloured according to categorisations of hydrophobic, neutral and hydrophilic types according to the free energy of insertion biological scale [36]. *Grey* represents hydrophilic amino acids that were found to have a positive Δ*G*_app_, and *blue* represents hydrophobic residues with a negative Δ*G*_app_, *purple* denotes negative residues and positive residues are coloured in *orange*. The abundances of key residues are labelled

The hydrophobic nature of the lipid bilayer membrane implies that, generally, charged residues should be rare within TMHs. For acidic residues, even the location in the sequence vicinity of TMHs should be disfavoured because of the negatively charged head groups of lipids directed towards the aqueous extracellular side or the cytoplasm. In agreement with the biophysically justified expectations, the statistical data confirm that acidic residues are especially rare in TMHs and their flanking regions. In Fig. 1, where we plot the total abundance of all amino acid types in single-pass TMHs and multi-pass TMHs (including their ±5 flanking residues), acidic residues were found to be amongst the rarest amino acids both in UniHuman and ExpAll.

The effect is most pronounced in single-pass transmembrane proteins (Fig. 1a). There are only 666 glutamates (just 1.24% of all residues) and 560 aspartates (1.05% respectively) amongst the total set of 53,238 residues comprising 1705 TMHs and their flanks. Within just the TMH regions, there are 71 glutamates (0.20% of all residues in TMHs and flanks) and 58 aspartates (0.16% respectively). This cannot be an artefact of UniProt TMH assignments since this feature is repeated in ExpAll. There are only 582 glutamates (1.22%) and 520 aspartates (1.09%) amongst the 47,568 residues involved. Within the TMH itself, there are 64 glutamates (0.19%) and 69 aspartates (0.21%). In both cases, the negatively charged residues represent the ultimate end of the distribution. Note that acidic residues are rare even compared to positively charged residues, which are about three to four times more frequent. On a much smaller dataset of single-spanning transmembrane proteins, Nakashima and Nishikawa [51] made similar compositional studies. To compare, they found 0.94% glutamate and 0.94% aspartate within just the TMH region (these values are very similar to ours from TMHs with small flanks; apparently, they used more outwardly defined TMH boundaries), but the content of each glutamate and aspartate within the extracellular or cytoplasmic domains is larger by an order of magnitude, between 5.26% and 9.34%. These latter values tend to be even higher than the average glutamate and aspartate composition throughout the protein database (5–6% [51]).

In the case of multi-pass transmembrane proteins (Fig. 1c and d), glutamates and aspartates are still very rare in TMHs and their ±5 residue flanks (1.94% and 1.92% from the total of 377,207 in the case of UniHuman, 1.79% and 1.70% from the total of 454,700 in the case of ExpAll). Yet, their occurrence is similar to those of histidine and tryptophan and, notably, acidic residues are only about ~1.5 times less frequent than positively charged residues. The observation that acidic residues are more suppressed in single-pass TMHs compared with multi-pass TMHs is statistically significant. In Table 1, the acidic residues are counted in the helices (excluding flanking regions) belonging to either multi-pass or single-pass helices. Indeed, single-pass helices appear to tolerate negative charge to a far lesser extent than multi-pass helices, as the data in the top two rows of Table 1 indicate (for datasets UniHuman and ExpAll). The trend is strictly observed throughout subcellular localisations (rows 3–5 in Table 1) and taxa (rows 6–10). Statistical significance (*P* ≤ 0.001) is found in all but six cases. These are UniEcoli (D + E, D, E), UniArch (D + E, E) and UniFungi (E). The problem is, most likely, that the respective datasets are quite small. Notably, the difference between single- and multi-pass TMHs is greatest in UniPM; here, TMHs from multi-pass proteins have on average 0.400 negative residue per helix, whereas single-pass TMHs contained just 0.039 (*P* = 3.86e-28).

### Amino acid residue distribution analysis reveals a “negative-not-inside/negative-outside” signal in single-pass TMH segments

**Fig. 2:**
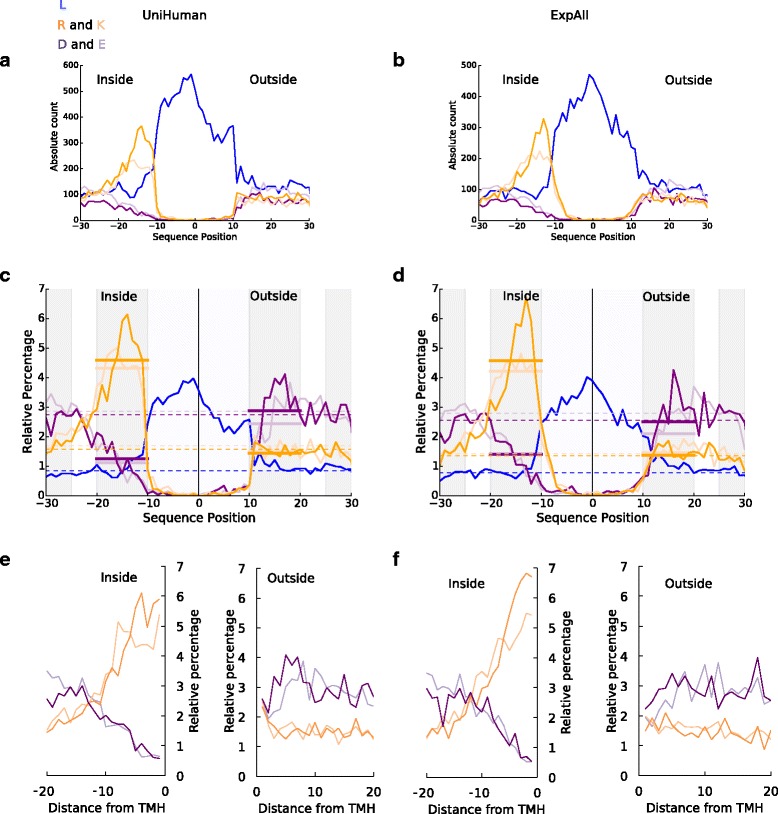
Relative percentage normalisation reveals a negative-outside bias in TMHs from single-pass protein datasets. All flank sizes were set at up to ±20 residues. We acknowledge that all values, besides the averaged values, are discrete, and connecting lines are illustrative only. On the *horizontal axes* (**a**–**d**) are the distances in residues from the centre of the TMH, with the negative numbers extending towards the cytoplasmic space. For **e** and **f**, the *horizontal axis* represents the residue count from the membrane boundary with negative counts into the cytoplasmic space. Leucine, the most abundant non-polar residue in TMHs, is in *blue*. Arginine and lysine are shown in *dark* and *light orange* respectively. Aspartic and glutamic acid are showing in *dark* and *light purple* respectively. **a** and **b** On the *vertical axis* is the absolute abundance of residues in TMHs from single-pass proteins from (**a**) UniHuman and (**b**) ExpAll. Note that no clear trend can be seen in the negative residue distribution compared to the positive-inside signal and the leucine abundance throughout the TMH. **c** and **d** On the *vertical axis* is the relative percentage at each position for TMHs from single-pass proteins from (**c**) UniHuman and (**d**) ExpAll. The *dashed lines* show the estimation of the background level of residues with respect to the colour; an average of the relative percentage values between positions 25 to 30 and –30 to –25. The *thick bars* show the averages on the inner (positions –20 to –10) and outer (positions 10 to 20) flanks coloured to the respective amino acid type. Note a visible suppression of acidic residues on the inside flank when compared to the outside flank in single-pass proteins when normalising according to the relative percentage. **e** and **f** The relative distribution of flanks defined by the databases with the distance from the TMH boundary on the *horizontal axis*. The inside and outside flanks are shown in separate subplots. The colouring is the same as in **a** and **b**

The rarity of negatively charged residues is a complicating issue when one studies their distribution along the sequence positions of TMHs and their flanks. For UniHuman (Fig. 2a) and ExpAll (Fig. 2b), we plotted the absolute abundance of aspartic acid, glutamic acid, lysine, arginine and leucine at each position (i.e., it scales as the equivalent fraction in the total composition of the alignment column). Note that the known preference of positively charged residues towards the cytoplasmic side is nevertheless evident. Yet, it becomes apparent that any bias in the occurrence of the much rarer acidic residues is overshadowed by fluctuations in the highly abundant residues such as leucine.

The trends become clearer if the occurrence of specific residues is normalised with the total number of residues of the given amino acid type in the dataset observed in the sequence region studied as shown for UniHuman in Fig. 2c and for ExpAll in Fig. 2d. For comparison, we indicated background residue occurrences (dashed lines calculated as averages for positions –25 to –30 and 25 to 30). The respective average occurrences in the inside and outside flanks (calculated from an average of the values at positions –20 to –10 and 10 to 20 respectively) are shown with wide lines.

The “positive-inside rule” becomes even more evident in this normalisation: Whereas the occurrence of positively charged residues is about the background level at the outside flank, it is about two to three times higher both for the UniHuman and the ExpAll datasets at the inside flank. Note that the background level was found to be 1.7% (lysine) and 1.6% (arginine) in UniHuman and 1.4% (lysine and arginine) in ExpAll. The inside flank average is 4.3% (lysine) and 4.6% (arginine) in UniHuman and 4.2% (lysine) and 4.6% (arginine) in ExpAll. The outside flank is similar to the background noise levels: about 1.4% (lysine) and 1.5% (arginine) in UniHuman and about 1.5% (lysine) and 1.4% (arginine) in ExpAll.

Most interestingly, a "negative-inside depletion" trend for the negatively charged residues is apparent from the distribution bias. The inside flank averages for glutamic acid were 1.1% and 1.4% in UniHuman and ExpAll respectively; for aspartic acid, 1.2% and 1.4% in UniHuman and ExpAll respectively. Meanwhile, the outside flanks for aspartic acid and glutamic acid occurrences were measured at 2.9% and 2.4% respectively in UniHuman, and in ExpAll, these values for aspartic acid and glutamic acid were found to be 2.5% and 2.1% respectively. Against the background level of aspartic acid (2.8% and 2.9% in UniHuman) and glutamic acid (2.6% and 2.9% in ExpAll), the inside flank averages were found to be about 2 to 3 times lower than the background level whilst the outside flank averages were comparable to the background level (Fig. 2c and d). Taken together, this indicates a clear suppression of negatively charged residues at the inside flank of single-pass TMHs and a possible trend for negatively charged residues occurring preferentially at the outside flank. This is not an effect of the flank definition selection since the trend remains the same when using the database-defined flanks without the context of the TMH (Fig. 2e and f). For UniHuman (Fig. 2e), the negative charge expectancy on the inside flank does not reach above 2% until position –10 (D) and position –11 (E), whereas, on the outside flank, both D and E start >2%. The same can be seen in ExpAll (Fig. 2f), where negative residues reach above 2% only as far from the membrane boundary as at position –9 (D) and position –7 (E) on the inside but exceed 2% beginning with positions 1 (D) and 3 (E) on the outside.

The observation of negative charge suppression at the inside flank, herein the “negative-inside depletion” rule, is statistically significant throughout most datasets in this study. The inside-outside bias was counted using the Kruskal-Wallis (KW) test comparing the occurrence of acidic residues within 10 residues of each TMH inside and outside the TMH (Table 2). We studied both the database-reported flanks as well as those obtained from central alignment of TMHs (see Methods). The null hypothesis (no difference between the two flanks) could be confidently rejected in all cases (*P* value < 0.001 except for UniBacilli), the sign of the *H* statistic (KW) indicating suppression at the inside and/or preference for the outside flank (except for UniArch). Most importantly, acidic residues were found to be distributed with bias in ExpAll (*P* value <3.47e-58) and in UniHuman (*P* value = 1.13e-93). Whereas with UniBacilli, the problem is most likely the dataset size, the exception of UniArch, for which we observe a strong negative inside rule, is more puzzling and indicates biophysical differences of their plasma membranes.

**Table 2:** Statistical significances for negative charge distribution skew on either side of the membrane in single-pass TMHs

Single-pass		Database-defined flanks				Flanks after central alignment			
Dataset	Helices	Negative residues		*H* statistic	*P* value	Negative residues		*H* statistic	*P* value
		Inside	Outside			Inside	Outside		
ExpAll	1544	848	1648	258.59	3.47E-58	735	1541	262.29	5.44E-59
UniHuman	1705	780	1922	421.53	1.13E-93	652	1865	501.86	3.74E-111
UniER	132	78	156	23.76	1.09E-06	76	150	21.62	3.33E-06
UniGolgi	206	60	240	104.45	1.61E-24	54	239	107.18	4.06E-25
UniPM	493	197	578	177.68	1.56E-40	161	569	215.18	1.02E-48
UniCress	632	314	450	18.23	1.96E-05	231	444	55.80	8.01E-14
UniFungi	729	449	631	28.15	1.12E-07	413	627	38.08	6.79E-10
UniBacilli	124	90	113	3.73	5.35E-02	86	106	2.53	1.12E-01
UniEcoli	54	32	77	17.24	3.30E-05	30	74	14.74	1.24E-04
UniArch	48	113	8	49.66	1.83E-12	96	7	45.62	1.43E-11

The “Helices” column refers to the total TMHs contained in each dataset (ExpALL, TMHs from TOPDB [50]; UniHuman, human representative proteome; UniER, human endoplasmic reticulum representative proteome; UniGolgi, human Golgi representative proteome; UniPM, human plasma membrane representative proteome; UniCress, *Arabidopsis thaliana* (mouse-ear cress) representative proteome; UniFungi, fungal representative proteome; UniBacilli, Bacilli class representative proteome; UniEcoli, *Escherichia coli* representative proteome; UniArch, Archaea representative proteome; see Methods for details). In the “Database-defined flanks” column, the “Negative residues” column refers to the total number of negative residues found in the ±10 flanking residues on either side of the TMH and does not include residues found in the helix itself. In the “Flanks after central alignment” column, the “Negative residues” column refers to the total number of negative residues found in the –20 to –10 residues and the +10 to +20 residues from the centrally aligned residues of the TMH. Unlike the other tables, the global averages are derived from the ±20 datasets. The Kruskal-Wallis scores were calculated for negative residues by comparing the number of negatively charged residues that were within the 10 inside residues and the 10 outside residues in either case

### Amino acid residue distribution analysis reveals a general negative charge bias signal in outside flank of multi-pass TMH segments: the negative-outside enrichment rule

**Fig. 3:**
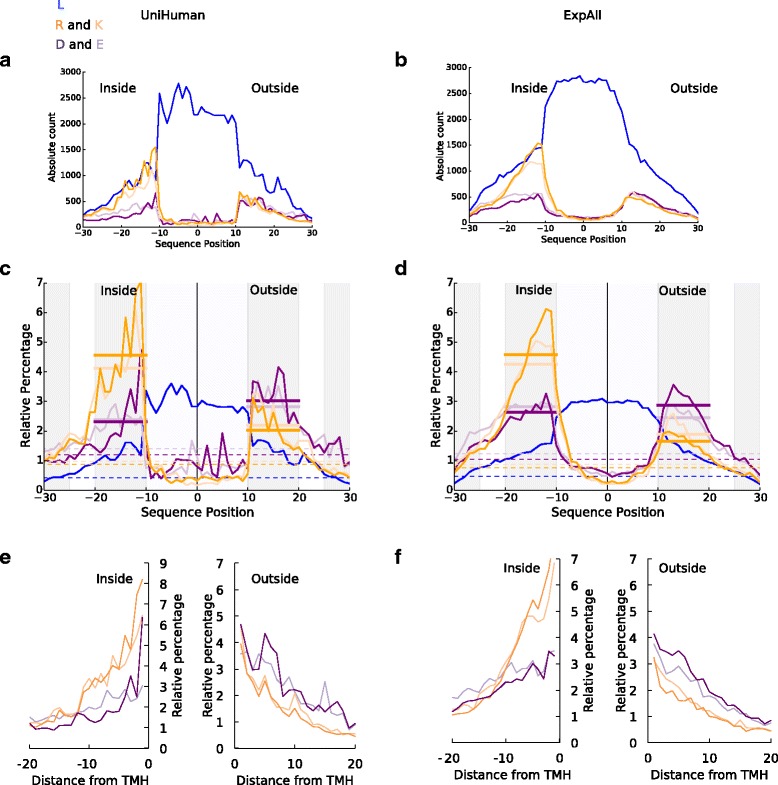
Negative-outside bias is very subtle in TMHs from multi-pass proteins. The meaning for the *horizontal axis* is the same as in Fig. 2, with the negative sequence position numbers extending towards the cytoplasmic space. Leucine is in *blue*. Arginine and lysine are shown in *dark* and *light orange* respectively. Aspartic and glutamic acid are shown in *dark* and *light purple* respectively. All flank sizes were set at up to ±20 residues. **a** and **b** On the *vertical axes* are the absolute abundances of residues from TMHs of multi-pass proteins from (**a**) UniHuman and (**b**) ExpAll. **c** and **d** On the *vertical axes* are the relative percentages at each position for TMHs from multi-pass proteins from (**c**) UniHuman and (**d**) ExpAll. As in Fig. 2c and d, the *dashed lines* show the estimation of the background level of residues with respect to the colour, and the *thick bars* show the averages on the inner and outer flanks coloured to the respective amino acid type. **e** and **f** The relative distribution of flanks defined by the databases with the distance from the TMH boundary on the *horizontal axis* for both the inside and outside flanks. The colouring is the same as in **a** and **b**

As a result of the rarity of negatively charged residues, any distribution bias is difficult to recognise in the plot showing the total abundance (or alignment column composition) of residues in multi-pass TMHs and their flanks from UniHuman (Fig. 3a) and ExpAll (Fig. 3b). Yet, as with single-pass helices, the dominant general leucine enrichment, as well as positive inside signal, can be identified with certainty. When the residue occurrence is normalised by the total occurrence of this residue type in the sequence regions studied (shown as a relative percentage at each position for multi-pass helices from UniHuman in Fig. 3c and e and ExpAll in Fig. 3d and f), the bias in the distribution of any type of charged residues becomes visible.

With regard to the positive-inside preference, positively charged residues have a background value of 2.0% for arginine and 2.2% for lysine in UniHuman, and 1.7% for arginine and 1.9% for lysine in ExpAll. At the inside flank, this rises to 4.6% for arginine and 4.1% for lysine in UniHuman and 4.6% for arginine and 4.2% for lysine in ExpAll. The mean net charge at each position was calculated for multi-pass and single-pass datasets from UniHuman and ExpAll (Additional file 1: Figure S1). The positive-inside rule clearly becomes visible, as the net charge has a positive skew approximately between residues –10 and –25. What is noteworthy is that the peaks found for single-pass helices were almost three times greater than those of multi-pass helices. For single-pass TMHs, the peak is +0.30 at position –15 in UniHuman and +0.31 at position –14 in ExpAll, whereas TMHs from multi-pass proteins had lower peaks of +0.15 at position –13 in UniHuman and +0.10 at position –14 in ExpAll. Thus, there is a positive charge bias towards the cytoplasmic side; yet, it is much weaker for multi-pass than for single-pass TMHs.

Notably, a "negative-outside enrichment" trend also can be seen from the distribution of the negatively charged residues, though with some effort (Table 3), as the effect is also weaker than in the case of single-pass TMHs. We studied the flanks under four conditions: (1) database-defined flanks without overlap between neighbouring TMHs, (2) flanks after central alignment of TMHs without flank overlap, (3) database-defined flanks but allowing overlap of flanks shared amongst neighbouring TMHs, (4) same as condition (2) but only the subset of cases where there is at least half of the required flank length at either side of the TMH. In UniHuman as calculated under condition (1), aspartic acid is lower on the inside flank (2.3%) than on the outside flank (3.0%). Glutamic acid is also lower at the inside flank (2.4%) than the 2.8% on the outside flank (Fig. 3c). Slight variations in defining the membrane boundary point do not influence the trend (compare Fig. 3c and e). We find that, in all studied conditions, the UniHuman dataset delivers statistical significances (*P* values: (1) 6.10e-34, (2) 5.43e-41, (3) 3.00e-57, (4) 5.60e-41), strongly supporting negative charge bias (inside suppression/outside preference; see Table 3).

**Table 3:** Statistical significances for negative charge distribution skew on either side of the membrane in multi-pass TMHs

A)												
Multi-pass					Database-defined flanks				Flanks after central alignment			
Dataset	IDs	Helices			Negative residues		*H* statistic	*P* value	Negative residues		*H* statistic	*P* value
		*n*	μ	σ	Inside	Outside			Inside	Outside		
ExpAll	2205	15,563	7.07	3.95	9709	9598	0.04	8.43E-01	9648	9659	0.35	5.56E-01
UniHuman	1789	12,353	6.93	3.20	7196	9164	147.50	6.10E-34	6740	8968	179.77	5.43E-41
UniER	155	898	5.85	3.20	630	584	0.44	5.08E-01	578	576	0.03	8.58E-01
UniGolgi	61	383	6.28	2.97	274	261	0.02	8.75E-01	266	259	0.09	7.65E-01
UniPM	427	3079	7.22	3.30	1945	2499	47.98	4.30E-12	1791	2440	64.42	1.01E-15
UniCress	507	3823	7.55	3.32	2567	2426	0.73	3.93E-01	2398	2433	1.11	2.93E-01
UniFungi	1338	8685	6.50	3.75	5560	5266	5.83	1.57E-02	5140	5214	0.00	9.62E-01
UniBacilli	140	822	5.94	3.98	470	468	0.07	7.92E-01	450	471	0.92	3.38E-01
UniEcoli	529	3888	7.39	3.76	1990	1902	0.26	6.07E-01	1875	1887	0.18	6.71E-01
UniArch	59	327	5.97	2.73	245	175	7.98	4.72E-03	235	181	7.08	7.81E-03
B)												
Multi-pass	Overlapping flanks				Database-defined viable* flanks							
Dataset	Negative residues		*H* statistic	*P* value	*N*	Negative residues		*H* statistic	*P* value			
	Inside	Outside				Inside	Outside					
ExpAll	11,969	12,615	22.54	2.05E-06	8808	6082	6916	59.93	9.81E-15			
UniHuman	8645	11,181	254.30	3.00E-57	8183	5169	6915	179.71	5.60E-41			
UniER	750	763	1.16	2.81E-01	516	398	441	3.16	7.55E-02			
UniGolgi	333	369	7.12	7.64E-03	195	162	186	3.00	8.30E-02			
UniPM	2319	3107	99.68	1.79E-23	1977	1343	1960	98.63	3.05E-23			
UniCress	3142	3298	9.21	2.41E-03	2110	1626	1741	6.40	1.14E-02			
UniFungi	6724	6814	0.46	4.96E-01	4581	3340	3411	0.41	5.22E-01			
UniBacilli	585	636	2.65	1.04E-01	382	230	306	12.73	3.61E-04			
UniEcoli	2574	2800	17.88	2.35E-05	1596	951	1114	16.57	4.69E-05			
UniArch	342	248	14.67	1.28E-04	132	120	104	0.28	5.97E-01			

The “Helices” column refers to the total TMHs contained in each dataset (ExpALL, TMHs from TOPDB [50]; UniHuman, human representative proteome; UniER, human endoplasmic reticulum representative proteome; UniGolgi, human Golgi representative proteome; UniPM, human plasma membrane representative proteome; UniCress, *Arabidopsis thaliana* (mouse-ear cress) representative proteome, UniFungi, fungal representative proteome; UniBacilli, Bacilli class representative proteome; UniEcoli, *Escherichia coli* representative proteome; UniArch, Archaea representative proteome; see Methods for details). In (A) the “Database-defined flanks” and in (B) the “Database-defined viable* flanks” and the “Overlapping flanks” columns, the “Negative residues” column refers to the total number of negative residues found in the ±10 flanking residues on either side of the TMH and does not include residues found in the helix itself. (A) In the “Flanks after central alignment” column, the “Negative residues” column refers to the total number of negative residues found in the –20 to –10 residues and the +10 to +20 residues from the centrally aligned residues with a maximum database defined flank length of 20 residues. The total number of proteins is given in the IDs column. The “Helices” column contains the total number of TMHs in the dataset (*n*), the average number of TMHs per protein in that population (*μ*) and the standard deviation of that average (*σ*). The Kruskal-Wallis scores were calculated for negative residues by comparing the number of negatively charged residues that were within 10 residues inside and 10 residues outside the helices

*Here, viable indicates that in each TMH used for both flanks either side of the TMH has a flank length of at least half the maximum allowed flank length, in this case 10 (viable length = 5)

Surprisingly, the result could not straightforwardly be repeated with the considerably smaller ExpAll. Under condition (1), we find with ExpAll that aspartic acid has a background level of 1.0%, an average of 2.6% on the inside flank and of 2.9% on the outside flank, but glutamic acid’s background is 1.2% but 2.8% on the inside flank and 2.5% on the outside flank. Statistical tests do not support finding a negative charge bias in conditions (1) and (2). Apparently, the problem is TMHs having no or almost no flanks at one of the sides. Statistical significance for the negative charge bias is detected as soon as this problem is dealt with — either by allowing extension of flanks overlap amongst neighbouring TMHs as in condition (3) or by excluding examples without proper flank lengths from the dataset as in condition (4). The respective *P* values under these conditions are 2.05e-6 and 9.81e-15.

The issues we had with ExpAll raised the question that sequence redundancy in the UniHuman set may have played a role. Therefore, we repeated all calculations but with UniRef50 instead of UniRef90 for mapping into sequence clusters (see the Methods section for details). We were surprised to see that harsher sequence redundancy requirements do not affect the outcome of the statistical tests in any major way. For the conditions (1)–(4), we computed the following *P* values: (1) 1.31e-28 (5940 negatively charged residues inside vs 7492 outside), (2) 1.38e-36 (5516 vs 7320), (3) 5.60e-53 (7089 vs 9233) and (4) 4.18e-41 (4232 vs 5730).

So, the amplifying effect of some subsets in the overall dataset on the statistical test that might be caused by allowing overlapping flanks (condition (3)) is not the major factor leading to the negative charge skew. Similarly, the trend is also not caused by sequence redundancy. Thus, we have learned that the negative charge bias does also exist in multi-pass transmembrane proteins but under the conditions that there are sufficiently long loops between TMHs. Bluntly stated: No loops equals to no charge bias. As soon as the loops reach some critical length, there are differences between single-pass and multi-pass TMHs with regard to occurrence and distribution of negative charges and the inside-suppression/outside-enrichment negative charge bias appears. Not only are there more negative charges within the multi-pass TMH itself (in fact, negative charges are almost not tolerated in single-pass TMHs; see Table 1), but also, there is a much stronger negative-outside skew in the TMHs of single-pass proteins than in those of multi-pass proteins.

### Further significant sequence differences between single-pass and multi-pass helices: distribution of tryptophan, tyrosine, proline and cysteine

**Fig. 4:**
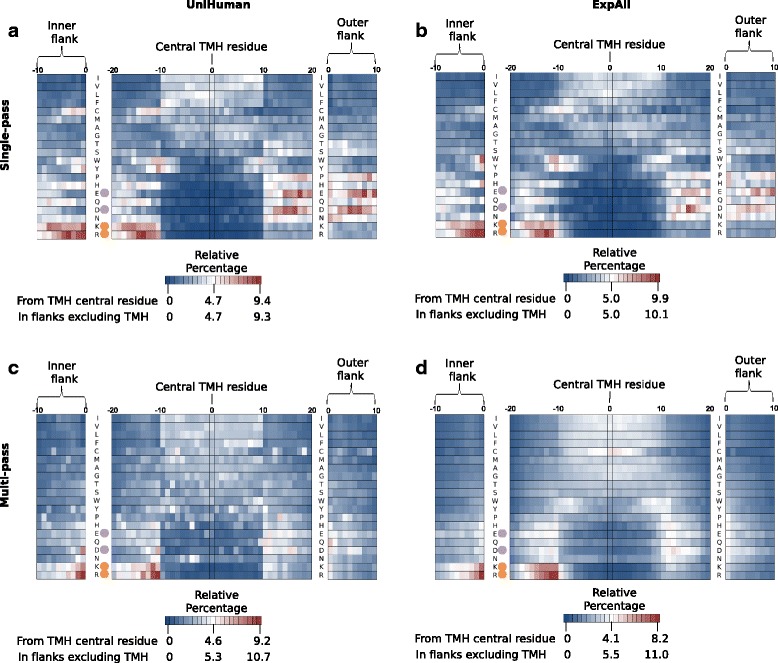
Relative percentage heatmaps from predictive and experimental datasets corroborate residue distribution differences between TMHs from single-pass and multi-pass proteins. The residue position aligned to the centre of the TMH is on the *horizontal axis*, and the residue type is on the *vertical axis*. Amino acid types are listed in order of decreasing hydrophobicity according to the Kyte and Doolittle scale [52]. The flank lengths in the TMH segments were restricted to up to ±10 residues. The scales for each heatmap are shown beneath the respective subfigure. The *darkest blue* represents 0% distribution, whilst the *darkest red* represents the maximum relative percentage distribution that is denoted by the keys in each subfigure, with *white* being 50% between “cold” and “hot”. The central TMH subplots extend from the central TMH residue, whereas the inner and outer flank subplots use the database-defined TMH boundary and extend from that position. **a** TMHs from the single-pass UniHuman dataset. **b** Single-pass protein TMHs from the ExpAll dataset. **c** TMHs from the proteins of the multi-pass UniHuman dataset. **d** TMHs from ExpAll multi-pass proteins. The general consistency in relative distributions of every residue type between single-pass and multi-pass of either dataset including flank/TMH boundary selection allows us to infer biological conclusions from these distributions that are independent of methodological biases used to gather the sequences. The only residue that displays drastically differently between the datasets is cysteine in multi-pass TMHs only. The most striking differences in distributions between residues from TMHs of single-pass and multi-pass proteins include a more defined Y and W clustering at the flanks, a suppression of E and D on the inside flank, a suppression of P on the inside flank and a topological bias for C favouring the inside flank

Amino acid residue profiles along the transmembrane segment and its flanks differ between single- and multi-pass TMHs also in other aspects. The relative percentages of all amino acid types (normalisation by the total amount of that residue type in the sequence segment) from single-pass helices of the UniHuman (Fig. 4a; from 1705 TMHs with flanks having 68,571 residues) and ExpAll (Fig. 4b; from 1544 TMHs with flanks having 60,200 residues) were plotted as a heatmap. The amino acid types were listed on the vertical axis according to Kyte and Doolittle hydrophobicity [52] in descending order.

In accordance with expectations, enrichment for hydrophobic residues in the TMH, for the positively charged residues on the inside flank as well as a distribution for the negative distribution bias, was found in both datasets. Additionally, the inside interfacial region showed consistent enrichment hotspots for tryptophan (e.g. 7.1% at position –11 in ExpAll, 6.2% at position –10 in UniHuman with flanks after central TMH alignment) and tyrosine (6.4% at –11 in ExpAll, 7.1% at –11 in UniHuman), and some preference can also be seen for the outer interfacial region (e.g. 5.2% at position 11 for tryptophan in ExpAll and 5.8% at position 10 for tryptophan in UniHuman), albeit the “hot” cluster of the outer flank covers fewer positions than that of the inner flank. Further, there is an apparent bias of cysteine on the inner flank and interfacial region (e.g. 5.5% at position –10 in ExpAll, 5.9% at position –11 in UniHuman) and a depression in the outer interfacial region and flank (up to a minimum of 0.3% in both ExpAll and UniHuman). Proline appears to have a depression signal on the outer flank. Note that, in a similar way to Figs. 2 and 3, the distributions of the flanks derived from centrally aligned TMHs are corroborated by the distributions from the database-defined TMH boundary flanks (see outside bands in Fig. 4a–d).

A similar heatmap was generated for UniHuman multi-pass TMHs (Fig. 4c; from 12,353 TMHs with flanks having 452,708 residues) and ExpAll multi-pass (Fig. 4d; from 15,563 TMHs with flanks having 535,599 residues). Whereas the heatmaps of Fig. 4a–c appear quite noisy, the plot for ExpAll multi-pass TMHs appears almost to have undergone Gaussian-like smoothing, thus, indicating the quality of this dataset. Tyrosine and tryptophan in the multi-pass case do not appear as enriched in the interfacial regions of single-pass TMHs from both UniHuman and ExpAll. Prolines are only suppressed in the TMH itself and are not suppressed in the outer flank as in the single-pass case but, indeed, are tolerated if not slightly enriched in the flanks.

### Hydrophobicity and leucine distribution in TMHs in single- and multi-pass proteins

**Fig. 5:**
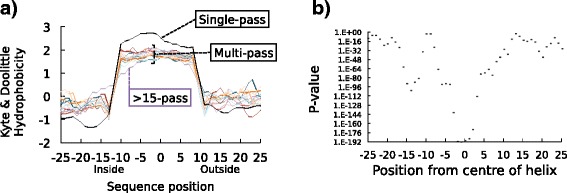
There is a difference in the hydrophobic profiles of TMHs from single-pass and multi-pass proteins. **a** The hydrophobicity of single-pass TMHs compared to multi-pass segments from the UniHuman dataset. The Kyte and Doolittle scale of hydrophobicity [52] was used with a window length of 3 to compare TMHs from proteins with different numbers of TMHs. This scale is based on the water-vapour transfer of free energy and the interior-exterior distribution of individual amino acids. The same datasets also had different scales applied (Additional file 2: Figure S2). The *vertical axis* is the hydrophobicity score, whilst the *horizontal axis* is the position of the residue relative to the centre of the TMH, with negative values extending into the cytoplasm. In *black* are the average hydrophobicity values of TMHs belonging to single-pass TMHs, whilst in *other colours* are the average hydrophobicity values of TMHs belonging to multi-pass proteins containing the same numbers of TMHs per protein. In *purple* are the TMHs from proteins with more than 15 TMHs per protein that do not share a typical multi-pass profile, perhaps due to their exceptional nature. **b** The Kruskal-Wallis test (*H* statistic) was used to compare single-pass windowed hydrophobicity values with the average windowed hydrophobicity value of every TMH from multi-pass proteins at the same position. The *vertical axis* is the logarithmic scale of the resultant *P* values. We can much more readily reject the hypothesis that hydrophobicity is the same between TMHs from single-pass and multi-pass proteins in the core of the helix and the flanks than the interfacial regions, particularly at the inner leaflet due to leucine asymmetry (Table 4)

Generally, we see in Fig. 4 that compositional biases appear more extreme in the single-pass case, particularly when it comes to polar and non-polar residues being more heavily suppressed and enriched. To investigate this observation, we calculated the hydrophobicity at each sequence position averaged over all TMHs considered (after having window-averaged over three residues for each TMH) using the Kyte and Doolittle hydrophobicity scale [52] (Fig. 5a) and validated using the White and Wimley octanol-interface whole residue scale [53], Hessa’s biological hydrophobicity scale [36] and the Eisenberg hydrophobic moment consensus scale [54] (Additional file 2: Figure S2). The total set of TMHs was split into 15 sets of membrane-spanning proteins (1 set containing single-pass proteins, 13 sets each containing TMHs from 2-, 3-, 4-… 14-transmembrane proteins and another of TMHs from proteins with 15 or more transmembranes). In Fig. 5b, we show the *P* value at each sequence position by comparing the respective values from multi-pass and single-pass TMHs using the two-sample *t* test (Fig. 5b). Strikingly, the inside flank of the single-pass TMHs is much more hydrophilic (e.g. see the Kyte and Doolittle score = –1.3 at position –18) than that of multi-pass TMHs (*P* value = 5.64e-103 at position –14). Most likely, the positive-inside rule along with the interfacial clustering of tryptophan and tyrosine contribute to a strong polar inside flank in single-pass helices that is not present in multi-pass helices *en masse*. Further, multi-pass TMHs cluster remarkably closely within the transmembrane core; the respective hydrophobicity is apparently not dependent on the number of TMHs in a given multi-pass transmembrane protein. On average, single-pass TMHs are more hydrophobic in the core than multi-pass TMHs (*P* value < 1.e-72 within positions –5…5 and *P* value = 5.92e-190 at position 0). On the other hand, hydrophobicity differences between TMHs from single- and multi-pass proteins fade somewhat at the transition towards the flanks (*P* value = 1.85e-4 at position –10, and *P* value = 3.35e-31 at position 10).

Leucine is the most abundant residue in TMHs (Fig. 1) and is considered one of the most hydrophobic residues by all hydrophobicity scales. Therefore, it plays a very influential role in TMH helix-helix and lipid-helix interactions in the membrane and recognition by the insertion machinery. When looking at the difference in the abundance of leucine between the inner and outer halves, we find that TMHs from single-pass proteins have a trend to contain more leucine residues at the cytoplasmic side of TMHs (see Figs. 2 and 4).

**Fig. 6:**
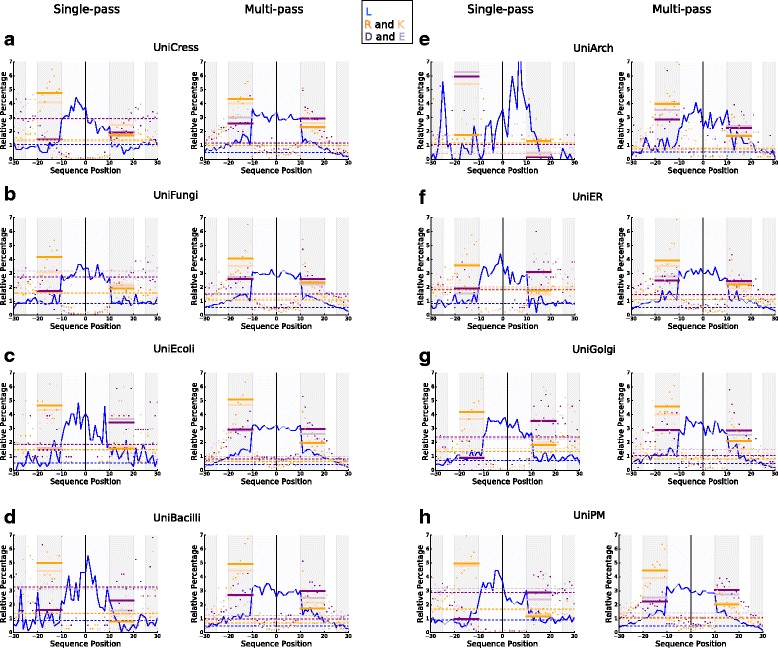
Comparing charged amino acid distributions in TMHs of multi-pass and single-pass proteins across different species and organelles. The relative percentage distribution of charged residues and leucine was calculated at each position in the TMH with flank lengths of ±20 in different datasets. The distributions are normalised according to relative percentage distribution. Aspartic acid and glutamic acid are shown in *dark purple* and *light purple* respectively. Leucine, the most abundant non-polar residue in TMHs, is in *blue*. Arginine and lysine are shown in *orange*. TMHs from single-pass proteins are on the *left* and TMHs from multi-pass proteins are on the *right* for different taxonomic datasets: **a** UniCress, **b** UniFungi, **c** UniEcoli, **d** UniBacilli, **e** UniArch, and different organelles: **f** UniER, **g** UniGolgi, **h** UniPM. As a trend, the negative-outside skew is more present in TMHs from single-pass proteins than multi-pass proteins (Tables 2 and 3). Another key observation is that in single-pass TMHs there is a propensity for leucine on the inner over the outer leaflet (Table 4)

This trend is statistically significant for TMHs in many biological membranes (Table 4, Fig. 6). In the most extreme case of UniCress (single-pass), we see 49% more leucine residues on the inside leaflet than the outside leaflet (*P* value = 5.41e-24). This contrasts with UniCress (multi-pass), in which the skew is far weaker, albeit yet statistically significant. There are 6% more leucine residues at the inside half (*P* value = 2.08e-4). The trend of having more leucine residues at the cytoplasmic half of the TMH is observed for all datasets (both single- and multi-pass) except for UniArch (single-pass). The phenomenon is statistically significant with *P* values < 1.e-3 for ExpAll, UniHuman, UniPM and UniCress (both single- and multi-pass). As with negative charge distribution, UniArch presents a reversed effect compared to other single-pass protein datasets with a 57% reduction in leucine on the inside leaflet compared to the outside leaflet (*P* value = 7.25e-6). However, leucines of TMHs from UniArch multi-pass proteins have no discernible preference for the inside leaflets (4% more on the inside leaflet, *P* value = 0.625).

**Table 4:** Leucines at the inner and outer leaflets of the membrane in TMHs

Dataset	Single-pass					Multi-pass				
	Inside	Outside	Percentage	*H* statistic	*P* value	Inside	Outside	Percentage	*H* statistic	*P* value
ExpAll	4020	3403	118.13	40.07	2.44E-10	27,986	27,008	103.62	14.13	1.70E-4
UniHuman	4982	3697	134.76	193.02	6.99E-44	25,199	22,365	112.67	195.24	2.29E-44
UniER	359	297	120.88	8.41	3.72E-3	1863	1764	105.61	3.98	4.61E-2
UniGolgi	604	513	117.74	10.74	1.05E-3	753	677	111.23	5.61	1.79E-2
UniPM	1485	1006	147.61	98.90	2.65E-23	6221	5577	111.55	35.21	3.00E-9
UniCress	1495	1005	148.76	102.05	5.41E-24	6491	6099	106.43	13.76	2.08E-4
UniFungi	1389	1308	106.19	3.41	6.48E-2	14,505	14,099	102.88	6.74	9.41E-3
UniBacilli	260	251	103.59	0.03	8.72E-1	1488	1335	111.46	7.59	5.89E-3
UniEcoli	130	100	130.00	2.78	9.53E-2	7251	6975	103.96	5.92	1.50E-2
UniArch	51	118	43.22	20.13	7.25E-6	636	612	103.92	0.24	6.25E-1

The statistical results when comparing the number of leucine residues from the inner and outer leaflets in each protein in the dataset. The number of helices per dataset can be found in Table 1. The Kruskal-Wallis test scores (*H* statistics) were calculated for leucine residues by comparing the number of leucine residues that were in the inner half of the leaflet with those in the outer half of the leaflet of the database-defined TMH

### A negative-outside (or negative-not-inside) signal is present across many membrane types

We explored the presence of amino acid residue compositional skews described above for human transmembrane proteins for those in other taxa and also specifically for human proteins with regard to membranes at various subcellular localisations. Acidic residues for TMHs from single-pass and multi-pass helices were plotted according to their relative percentage distributions (of the total amount of this residue type in the respective segment) for five taxon-specific datasets: UniCress (Fig. 6a), UniFungi (Fig. 6b), UniEcoli (Fig. 6c), UniBacilli (Fig. 6d), UniArch (Fig. 6e), and for three organelle-specific datasets: UniER (Fig. 6f), UniGolgi (Fig. 6g), UniPM (Fig. 6h).

For single-pass proteins in all taxon-specific datasets (with the exception of UniArch), there are more negative residues at the outside than at the inside. The skew is statistically significant (see Table 2, *P* < 0.001) except for UniBacilli. However, despite statistical significance found for UniFungi (*P* value = 1.12e-7 for database-defined and *P* value = 6.79e-10 for flanks after central alignment; Table 2), the trend is not very strong in this case (Fig. 6b). Whereas the skew is just a suppression of negatively charged residues at the inside flank for ExpAll and UniHuman (as well as in UniCress), the bias observed for UniEcoli also involves a negative charge enrichment at the outside flank. In the case of UniArch (Fig. 6e), we see a negative inside preference that is 6.0% in the case of aspartic acid and 6.3% for glutamic acid (not shown), with much lower values close to 0% on the outside. Whilst the difference is statistically significant for both TMHs (Table 2) from single-pass proteins (*P* value = 1.83e-12 and *P* value = 1.43e-11 for two versions of flank determination) and multi-pass proteins (*P* values 4.72e-3, 7.81e-3, 1.28e-4 for three versions of flank determination, see Table 3A and B), the distribution along the position axis is heavily fluctuating, perhaps as a result of the small size of the dataset. However, one can assuredly assign a “negative-inside” tendency to the flanking regions of Archaean TMHs.

In the human organelle datasets, we see trend shifts at different stages in the secretory pathway. In UniER, there is an enrichment of negative charge on the outside flank of 1–1.5% that is comparable to the magnitude of the positive inside signal. In UniGolgi, there is a suppression of negatively charged residues on the inside flank as well as an enrichment on the inside flank resulting in ~2% distribution difference. For UniPM, there is a negative-inside suppression (but no outside enrichment) as well as a positive-inside signal. All observed trends are statistically significant (see Table 2, *P* < 1.e-5).

For multi-pass TMH proteins, we either see the same trends but in a weaker form, or no skews are observed at all, as inspection of the graphs in Fig. 6 shows. For datasets UniER, UniGolgi, UniCress, UniFungi and UniBacilli, the hypothesis of equal distribution of negatively charged residues cannot be rejected (*P* value > 0.001, see Table 3); thus, a skew is statistically non-significant. Although UniPM has a statistically significant bias (*P* value < 4.30e-12, Table 3), the trends are more subtle and most present for aspartic acid of UniPM. We see many more negative and positive charges tolerated within the multi-pass TMHs themselves throughout all datasets (Table 1). We note that there is a positive-inside rule for all multi-pass datasets studied herein.

To conclude, we find that negative charge bias distribution is a feature of single-pass protein TMHs that is present across many membrane types, and it can have the form of a negative charge suppression at the inside flank or an enrichment of those charges at the outside flank.

### Amino acid compositional skews in relation to TMH complexity and anchorage function

**Fig. 7:**
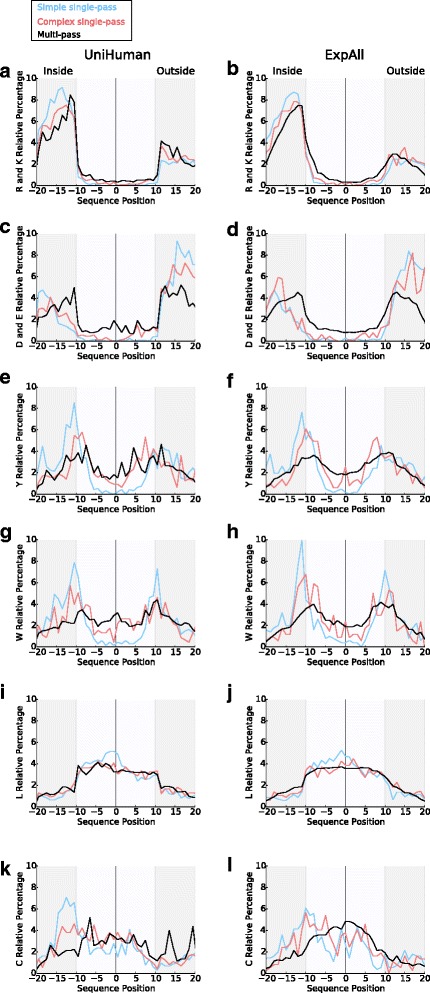
Comparing the amino acid relative percentage distributions of simple and complex TMHs from single-pass proteins and TMHs from multi-pass proteins. TMSOC was used to calculate which single-pass TMHs were complex and which were simple from ExpAll and UniHuman datasets. Simple TMHs are typically anchors without necessarily having other functions (Wong et al. [5]). The relative percentages from single-pass simple (shown in *light blue*), single-pass complex (*red*), and multi-pass protein TMHs (*black*) were plotted for (**a**, **c**, **e**, **g**, **i** and **k**) UniHuman and (**b**, **d**, **f**, **h**, **j** and **l**) ExpAll for (**a** and **b**) positive residues, (**c** and **d**) negative residues, (**e** and **f**) tyrosine, (**g** and **h**) tryptophan, (**i** and **j**) leucine and (**k** and **l**) cysteine. The slopes are statistically compared in Tables 5 and 6, and as a trend, the profiles of complex TMHs are more similar to multi-pass TMH profiles than simple TMHs are to multi-pass TMHs

In previous work, we studied the relationship of TMH composition, sequence complexity and function [5–7] and concluded that simple TMHs are more probably responsible for simple membrane anchorage, whereas complex TMHs have a biological function beyond just anchorage. We wished to see how the skews observed in this work relate to that classification. Therefore, the single-pass TMHs from UniHuman and ExpAll were separated into subsets of simple, twilight and complex TMHs using the webserver Transmembrane helix: Simple Or Complex (TMSOC) [6, 7]. The relative percentages of eight residue types (L, D, E, R, K, Y, W, C; normalisation with the total amount of residues of that amino acid type in all sequence segments considered) were plotted along the sequence position for simple and complex helices (Fig. 7). Of UniHuman single-pass proteins, there were 889 records with simple TMHs and 570 with complex TMHs (Fig. 7a). In ExpAll, 769 TMHs from single-pass proteins were simple TMHs and 570 were complex TMHs (Fig. 7b).

It is visually apparent (Fig. 7) that there are (1) stronger skews and more inside-outside disparities in simple single-pass transmembranes than in complex single-pass transmembranes and (2) greater similarities between single-pass complex transmembrane regions and those from multi-pass proteins compared with simple single-pass transmembranes in comparison with either of the other two distributions. To examine the statistical significance of these observations, we compared the amino acid distributions (K, R, K + R, D, E, D + E, Y, W, L, C) across the range of TMHs with flank lengths ±10 residues using the Kolmogorov-Smirnov (KS), the Kruskal-Wallis (KW) and the χ^2^ statistical tests. The KS test scrutinises for significant maximal absolute differences between distribution curves, the KW test looks for skews between distributions and the χ^2^ statistical test checks the average difference between distributions. Calculations were carried out over single-pass complex, single-pass simple and multi-pass TMH datasets from both ExpAll and UniHuman (for *P* values and Bahadur slopes, see Table 5 (dataset UniHuman) and Table 6 (dataset ExpAll)).

**Table 5:** Simple TMHs are less similar than complex TMHs to TMHs from multi-pass proteins in UniHuman

Residues	*P* values for χ2			Bahadur slopes for χ2		
	Simple-vs-complex	Simple-vs-multi	Complex-vs-multi	Simple-vs-complex	Simple-vs-multi	Complex-vs-multi
R	3.20E-06	7.38E-02	1.24E-01	6.61E-03	2.20E-03	1.27E-04
K	2.23E-03	4.99E-02	2.14E-01	3.99E-03	3.70E-03	1.18E-04
D	1.67E-09	3.06E-01	3.02E-01	3.34E-02	3.24E-03	1.20E-04
E	3.80E-07	2.34E-01	2.31E-01	1.81E-02	3.05E-03	1.36E-04
Y	3.86E-01	3.97E-01	2.11E-01	1.06E-03	1.47E-03	8.25E-05
W	3.77E-03	2.97E-01	3.84E-01	8.52E-03	2.73E-03	1.13E-04
L	3.59E-01	2.88E-01	3.21E-01	1.52E-04	3.92E-04	1.69E-05
C	6.44E-01	3.97E-01	3.41E-01	4.29E-04	1.29E-03	8.57E-05
R + K	2.19E-02	2.83E-01	2.52E-01	1.11E-03	6.33E-04	4.68E-05
D + E	1.47E-03	2.86E-01	2.79E-01	4.59E-03	1.49E-03	6.15E-05
	*P* values for Kolmogorov-Smirnov			Bahadur slopes for Kolmogorov-Smirnov		
	Simple-vs-complex	Simple-vs-multi	Complex-vs-multi	Simple-vs-complex	Simple-vs-multi	Complex-vs-multi
R	2.31E-01	3.57E-04	1.08E-02	7.66E-04	6.71E-03	2.76E-04
K	4.31E-02	2.18E-03	8.93E-01	2.06E-03	7.56E-03	8.68E-06
D	1.39E-01	5.02E-06	1.08E-02	3.26E-03	3.34E-02	4.52E-04
E	7.96E-02	1.58E-05	1.08E-02	3.10E-03	2.32E-02	4.20E-04
Y	7.96E-02	2.22E-02	2.31E-01	2.81E-03	6.07E-03	7.78E-05
W	2.31E-01	9.06E-04	4.31E-02	2.24E-03	1.58E-02	3.70E-04
L	2.31E-01	2.31E-01	5.31E-01	2.17E-04	4.61E-04	9.42E-06
C	1.39E-01	3.61E-01	3.61E-01	1.93E-03	1.42E-03	8.10E-05
R + K	7.96E-02	1.33E-04	7.96E-02	7.35E-04	4.48E-03	8.60E-05
D + E	4.31E-02	1.58E-05	4.98E-03	2.21E-03	1.31E-02	2.55E-04
	*P* values for Kruskal-Wallis			Bahadur slopes for Kruskal-Wallis		
	Simple-vs-complex	Simple-vs-multi	Complex-vs-multi	Simple-vs-complex	Simple-vs-multi	Complex-vs-multi
R	2.19E-01	5.06E-02	2.37E-01	7.92E-04	2.52E-03	8.79E-05
K	2.90E-01	1.33E-01	7.00E-01	8.11E-04	2.49E-03	2.73E-05
D	3.50E-01	1.81E-02	2.81E-01	1.74E-03	1.10E-02	1.27E-04
E	2.59E-01	5.65E-02	1.78E-01	1.65E-03	6.04E-03	1.60E-04
Y	6.03E-01	4.53E-01	4.41E-01	5.62E-04	1.26E-03	4.34E-05
W	4.19E-01	1.84E-01	5.70E-01	1.33E-03	3.81E-03	6.62E-05
L	6.37E-01	4.88E-01	9.77E-01	6.68E-05	2.25E-04	3.47E-07
C	5.00E-01	2.22E-01	9.62E-01	6.76E-04	2.10E-03	3.11E-06
R + K	1.87E-01	8.67E-02	4.08E-01	4.86E-04	1.23E-03	3.05E-05
D + E	1.68E-01	4.52E-02	1.91E-01	1.25E-03	3.68E-03	7.97E-05

The statistical results were gathered by comparing complex single-pass TMHs, simple TMHs from single-pass proteins and TMHs from multi-pass proteins in UniHuman. The abundance of different residues at each position when using the centrally aligned TMH approach was compared with several statistical tests (the Kolmogorov-Smirnov, Kruskal-Wallis and the χ^2^ statistical tests) and the Bahadur slope values of those results

**Table 6:** Simple TMHs are less similar than complex TMHs to TMHs from multi-pass proteins in ExpAll

Residues	*P* values for χ2			Bahadur slopes for χ2		
	Simple-vs-complex	Simple-vs-multi	Complex-vs-multi	Simple-vs-complex	Simple-vs-multi	Complex-vs-multi
R	5.10E-06	2.98E-01	5.10E-06	9.17E-03	1.61E-03	6.23E-05
K	2.35E-03	1.85E-01	2.35E-03	4.81E-03	3.88E-03	9.78E-05
D	2.61E-08	1.84E-01	2.61E-08	4.15E-02	7.90E-03	1.41E-04
E	2.38E-10	2.04E-01	2.38E-10	3.88E-02	7.08E-03	1.22E-04
Y	3.03E-01	3.11E-01	3.03E-01	2.01E-03	2.49E-03	5.51E-05
W	4.21E-03	4.29E-01	4.21E-03	1.11E-02	4.76E-03	6.46E-05
L	3.79E-01	3.04E-01	3.79E-01	2.28E-04	4.66E-04	1.50E-05
C	3.87E-01	2.52E-01	3.87E-01	1.75E-03	3.28E-03	1.48E-04
R + K	7.16E-04	2.52E-01	7.16E-04	2.80E-03	1.28E-03	3.76E-05
D + E	3.58E-05	2.94E-01	3.58E-05	1.03E-02	1.94E-03	4.90E-05
	*P* values for Kolmogorov-Smirnov			Bahadur slopes for Kolmogorov-Smirnov		
	Simple-vs-complex	Simple-vs-multi	Complex-vs-multi	Simple-vs-complex	Simple-vs-multi	Complex-vs-multi
R	3.61E-01	4.31E-02	3.61E-01	7.66E-04	7.79E-03	1.62E-04
K	4.31E-02	8.93E-01	4.31E-02	2.49E-03	1.05E-02	6.57E-06
D	1.39E-01	2.18E-03	1.39E-01	4.68E-03	3.61E-02	5.10E-04
E	5.31E-01	1.33E-04	5.31E-01	1.11E-03	2.81E-02	6.87E-04
Y	2.31E-01	9.06E-04	2.31E-01	2.47E-03	6.26E-03	3.30E-04
W	5.31E-01	4.98E-03	5.31E-01	1.29E-03	1.13E-02	4.04E-04
L	2.31E-01	2.31E-01	2.31E-01	3.45E-04	2.12E-03	1.85E-05
C	5.31E-01	3.61E-01	5.31E-01	1.16E-03	8.91E-04	1.09E-04
R + K	1.39E-01	2.31E-01	1.39E-01	7.61E-04	4.82E-03	4.00E-05
D + E	1.39E-01	9.06E-04	1.39E-01	1.99E-03	1.41E-02	2.80E-04
	*P* values for Kruskal-Wallis			Bahadur slopes for Kruskal-Wallis		
	Simple-vs-complex	Simple-vs-multi	Complex-vs-multi	Simple-vs-complex	Simple-vs-multi	Complex-vs-multi
R	4.37E-01	3.92E-01	4.37E-01	6.24E-04	2.52E-03	4.82E-05
K	3.83E-01	6.93E-01	3.83E-01	7.62E-04	2.88E-03	2.13E-05
D	4.49E-01	1.81E-01	4.49E-01	1.90E-03	1.06E-02	1.42E-04
E	7.64E-01	1.94E-01	7.64E-01	4.71E-04	9.05E-03	1.26E-04
Y	8.32E-01	3.36E-01	8.32E-01	3.09E-04	9.63E-04	5.15E-05
W	7.25E-01	1.36E-01	7.25E-01	6.53E-04	5.44E-03	1.52E-04
L	7.15E-01	7.95E-01	7.15E-01	7.90E-05	3.41E-04	2.90E-06
C	8.47E-01	9.54E-01	8.47E-01	3.05E-04	4.26E-05	5.06E-06
R + K	2.89E-01	5.13E-01	2.89E-01	4.79E-04	1.41E-03	1.82E-05
D + E	4.94E-01	2.07E-01	4.94E-01	7.11E-04	4.14E-03	6.29E-05

As in Table 5, the statistical results were gathered by comparing complex single-pass TMHs, simple TMHs from single-pass proteins and TMHs from multi-pass proteins; however, in this case only ExpAll is used. The abundance of different residues at each position when using the centrally aligned TMH approach was compared with several statistical tests (Kolmogorov-Smirnov, Kruskal-Wallis and the χ^2^ statistical tests) and the Bahadur slope values of those results

The many low *P* values in Tables 5 and 6 indicate significant differences between the three distributions studied. For the UniHuman dataset (Table 5), we find the most striking, significant differences between charged residue distributions (R, K, D, E) of simple and complex single-pass TMH + flank regions (χ^2^ *P* value < 2.23e-3 for single amino acid types). Similarly, simple single-pass TMH + flank segments differ significantly from multi-pass TMH + flank segments (KW test *P* values < 3.e-2 for R, K, D, E, Y, W amino acid types as well as for K + R and D + E). The trends are the same for the ExpAll dataset (Table 6): simple and complex single-pass TMH + flank regions differ in charged amino acid type distributions (χ^2^ *P* value < 4.21e-3 for all cases), as do simple single-pass and multi-pass ones (KW test *P* values < 5.e-2 for R, D, E, Y, W amino acid types and D + E).

Whereas *P* value tests for significant differences between distributions depend strongly on the amount of data, the more informative Bahadur slopes that measure the distance from the zero hypothesis are independent of the amount of data [55–57]. As we can see in Tables 5 and 6, the absolute Bahadur slopes for the simple single-pass to multi-pass comparison are always larger (even by at least an order of magnitude): (1) for all three statistical tests applied (χ^2^, KS and KW), (2) for all amino acid types, for K + R and E + D and (3) for both datasets UniHuman and ExpAll. Thus, complex single-pass TMH + flanks have compositional properties that are indeed very similar to those of multi-pass ones (which are known to have a large fraction of complex TMHs [6, 7]). This strong evidence implies that the actual issue is not so much about single- and multi-pass TMH segments but between simple and complex TMHs: The first are exclusively guided by the anchor requirements, whereas the latter have more complex restraints to fulfil.

Several distribution features of simple TMHs from single-pass proteins, when compared to complex TMHs from single-pass proteins and TMHs from multi-pass proteins, that contribute to the statistical differences (Fig. 7) are especially notable. There is a more pronounced trend for positively charged residues and tyrosine to be preferentially located on the inside flanks and for negatively charged residues to be on the outside flanks. The symmetrical peaks in the percentage distribution of tyrosine in complex single-pass TMHs are more akin to multi-pass TMHs, whereas in simple TMHs the distribution resembles a more typical single-pass helix (compare with Fig. 3). Furthermore, the depression of charged residues within the TMH itself is strongest in simple single-pass TMHs.

To emphasise, tryptophan is essentially not tolerated within the simple TMHs, and there are higher peaks of tryptophan occurrence at either flank. We also see a strong inside skew for leucine clustering within the core of simple TMHs which is not present in the “flatter” distributions of complex single-pass TMHs and TMHs from multi-pass proteins.

There is obviously a cysteine-inside preference for simple, single-pass TMHs but less in complex, multi-pass TMHs (Fig. 7). This conclusion is contrary to that of a previous study [51], but that deduction was drawn from a much smaller dataset of 45 single-pass TMHs and 24 multi-pass transmembrane proteins.

## Discussion

### The “negative-not-inside/negative-outside” skew in TMHs and their flanks is statistically significant

We have seen that, consistently throughout the datasets, there is a trend for generally rare negatively charged residues to prefer the outside flank of a TMH rather than the inside (and to almost completely avoid the TMH itself), be it by suppression on the inside and/or enrichment on the outside. The trend is much stronger in single-pass protein datasets than in multi-pass protein datasets. However, as we have elaborated, the real crux of the bias appears to be associated with the TMH being simple or complex [6, 7] and, thus, whether or not the TMH has a role beyond anchorage. The existence of this bias has implications for topology prediction of proteins with TMHs, engineering membrane proteins and also for models of protein transport via membranes and protein-membrane stability considerations.

It should be noted that the controversy in the scientific community about the existence of a negative charge bias at TMHs was mainly with regard to multi-pass transmembrane proteins. Despite having access to much larger, better annotated sequence datasets and many more three-dimensional (3D) structures than our predecessors, we also had our share of difficulties here (see the Results section titled: Amino acid residue distribution analysis reveals a general negative charge bias signal in outside flank of multi-pass TMH segments: the negative-outside enrichment rule and Table 3). The straightforward approach results in inconclusive statistical tests if datasets become small (for example, if selections are restricted to subcellular localisations or 3D structures or if very harsh sequence redundancy criteria are applied) and, especially, if TMHs with very short or no flanks are included. Therefore, in the case of multi-pass proteins, we studied flanks as taken from the transmembrane boundaries in the databases under several conditions: (1) without allowing flank overlap between neighbouring TMHs, (2) as a subset of (1) but with requiring some minimal flank length at either side and (3) with overlapping flanks. We also studied flanks after central alignment of TMHs and assuming standardised TMH length. Multi-pass TMHs (without overlapping flanks) do not show statistically significant negative charge bias under condition (1) but, apparently, because of many TMHs without any or super-short flanks, at least at one side. Significance appears as soon as subsets of TMHs with flanks at both sides are studied. Not surprisingly, there is no charge bias if there are no flanks in the first place. It is perhaps worth noting that the results from multi-pass TMHs with overlapping flanks may involve amplification of skews since this involves multiple counting of the same residues. Given the redundancy threshold of UniRef90, we cannot rule out that these statistical skews are the result of a trend from only a small subgroup of TMPs which is being amplified. Hence, we also needed to observe if these same observed biases were true in condition (2), which is indeed the case.

As the "negative-not-inside/negative-outside” skew is widely observed amongst varying taxa and subcellular localisations with statistical significance, it appears, at least to a certain extent, to be caused by physical reasons and to be associated with the background membrane potential. Several earlier considerations and observation support this thought: (1) Firstly, a concert between the negative and positive charge on the TMH flanks drives anchorage and the direction of insertion of engineered TMHs [29, 44]. (2) Secondly, the inner leaflet of the plasmalemma tends to be more negatively charged [58]. Specifically, phosphatidylserine was found to distribute in the cytosolic leaflets of the plasma membrane, and it was found to electrostatically interact with moderately positive-charged proteins enough to redirect the proteins into the endocytic pathway [59]. The negative charge of proteins at the inside of the plasma membrane would decrease the anchoring potency of the TMH via electrostatic repulsion. (3) Thirdly, in membranes that maintain a membrane potential, there are inevitably electrical forces acting on charged residues during chain translocation, as this influences the translocon machinery when orienting the TMH. Therefore, it is no surprise that we see an inside-outside bias for negatively charged residues that is opposite to the one for positively charged residues. The negative charges in TMH residues have been shown to experience an electrical pulling force as they pass through the bacterial SecYEG translocon import [42, 43]. Also, they are known to be involved in intra-membrane helix-helix interactions [60]. For example, aspartic acid and glutamic acid can drive efficient di- or trimerisation of TMHs in lipid bilayers and, furthermore, aspartic acid interactions with neighbouring TMHs can directly increase insertion efficiency of marginally hydrophobic TMHs via the Sec61 translocon [60]. In support of this, less acidic residues are found in single-pass TMHs, amongst which only some will undergo intra-membrane helix-helix interactions. As the mutation studies have shown negative charge as a topological determinant [35], it is perhaps no surprise that we observe a skew in negatively charged residues in a similar manner to the skew in positively charged residues.

Whereas the "negative-not-inside/negative-outside” skew is observed for distantly related eukaryotic species, and it is also present in Gram-negative bacteria such as *E. coli*, this sequence pattern was not observed for the Gram-positive bacteria, in which there is no observable bias. In contrast, Archaea have a statistically significant “negative-inside” propensity both for single- and multi-pass TM proteins. It is known that Archaea have remarkably different membranes compared to other kingdoms of life due to their extremophile adaptations to stress [61]. Whilst it is unclear why negative charge is distributed so differently in UniArch compared to the other taxonomic datasets, one must appreciate that a much more nuanced approach would be needed to draw formal conclusions about Archaea, which current databases cannot provide due to the relatively limited information and annotation of Archaean proteomes.

### Methodological issues made previous studies struggle to identify negatively charged skews with statistical significance

Whereas the influence of a negative charge bias in engineered proteins with transmembrane regions on the direction of insertion into the membrane was solidly established [35, 39, 40, 45, 62], the search for the negative charge distribution pattern in the statistics of sequences of transmembrane proteins from databases failed to find significance for the expected negative charge skew [9, 13, 14, 16, 31, 45].

Generally speaking, the datasets from previous studies have been considerably smaller compared with those in our work (only Sharpe et al. had a similar order of magnitude [9]), especially those with experimental information about 3D structure and membrane topology that we used for validation. And they might not have had the luxury of using UniProt’s improved TRANSMEM consensus annotation based on a multitude of transmembrane prediction methods and experimental data, but this is also not the major issue. We found that there are other factors that are critical for observing sequence bias such as negative charge skew in the case of TMHs:

1. Acidic residues are rare near and within TMHs, and biases in their distribution are easily blurred by minor fluctuations of much more frequent amino acid types, most notably leucine. Therefore, the method of normalisation is critical. We have shown that normalising by the total amount of residues of the amino acid type studied within the sequence region under consideration is appropriate to answer the question of where to find a negatively charged residue if there is any at all (called “relative percentage” in this work).
2. The alignment of the TMHs is critical. It was common practice to align the TMH according to the most cytosolic residue [9], although it is known that the membrane/cytosol boundary of the TMH is not well defined (and the exact boundary is even less well understood at the non-cytosolic side). Aligning the transmembrane regions and their flanks from the centre of the TMH was first proposed by Baeza-Delgado et al. [13]. Since we know now that acidic residues are often suppressed in the cytosolic flank and within the TMH, this implies that the few acidic residues found in the cytosolic interface would appear more comparable to those in the poorly defined non-cytosolic interface, as the respective residues are spread over more potential positions, diminishing any observable bias.
3. We find that separation into single- and multi-pass transmembrane datasets (or, even better, simple and complex transmembranes [6, 7]) is critical to study the inside/outside bias. As many TMHs in multi-pass transmembrane proteins have essentially no flanks or very short flanks if the condition of non-overlap is applied to flanks of neighbouring TMHs, this might also obscure the observation of the negative charge bias. If there are no flanks, then there will be no residue distribution bias in these flanks. The problem can be alleviated by either studying only subsets with minimal flank lengths on both sides (although datasets might become too small for statistical analysis) or by allowing flank overlaps between neighbouring TMHs.
4. This classification is even more justified in the light of previous reports about the “missing hydrophobicity” in multi-pass TMHs [36, 63–65]. Otherwise, the distribution bias well observed amongst the exclusive anchors could be lost to noise. This addresses the more biologically contextualised issue that there are different evolutionary pressures on different types of TMHs. The negative charge skew is most pronounced for dedicated anchors frequently found with simple TMHs typically observed in single-pass TM proteins. These TMHs are pressured to exhibit residue biases that may aid anchorage in a topologically correct manner. Complex TMHs, typically within multi-pass membrane proteins that have a function beyond anchorage, comply with a multitude of structural and functional constraints, and the negative charge skew is just one of them.

The most representative precedent papers are those of Sharpe et al. [9] from 2010 (with 1192 human and 1119 yeast single-pass TMHs), Baeza-Delgado et al. [13] (with 792 TMHs mixed from single- and multi-pass TM proteins) and Pogozheva et al. [16] (TMHs from 191 mixed from single- and multi-pass TM proteins with structural information), both from 2013. Whereas the first analysis would have benefitted from the central alignment approach and the first two studies from another normalisation as described above, the third study did come close to our findings. To note, their dataset mixed with single- and multi-pass proteins was too small for revealing the negative charge bias with significance; yet, they observed total charge differences at either side of the membrane varying for both single- and multi-pass proteins. Membrane asymmetry due to positively charged residues occurring more frequently on the cytosolic side causes net charge unevenness at both sides of the membrane. This observation has been known to correlate with orientation for decades [12, 13, 60]. Our data show that the negative charge skew contributes to this asymmetry.

### There are differences in charged amino acid residue biases in TMH flanks through each stage of the secretory pathway

Here, we observe differences throughout subcellular locations along the secretory pathway. We found that negative charges are enriched at the outside flank (in the ER), both enriched outside and suppressed inside for the Golgi membrane and suppressed on the inside flank in the plasma membrane (PM). It has been suggested that the leaflets of different membranes have different lipid compositions throughout the secretory pathway [66], and this has led to general biochemical conservation in terms of TMH length and amino acid composition in different membranes [9, 16].

Lipid asymmetry in the Golgi and PM (in contrast to the ER) has been known for more than a decade [67, 68]. To note, the Golgi and PM have lipid asymmetry with sphingomyelin and glycosphingolipids on the non-cytosolic leaflet and phosphatidylserine and phosphatidylethanolamine enriched in the cytosolic leaflet. Although the ER is the main site for cholesterol synthesis, it has markedly low concentrations of sphingolipids [69]. The Golgi synthesises sphingomyelin, a lipid not present in the ER, but present in both the Golgi [70] and in the PM [71, 72]. The PM is also enriched with densely packed sphingolipids and sterols [73]. Another factor influencing the sequence patterns of TMHs and their flanking regions along the secretory pathway appears to be the variation in membrane potentials [74–76].

### Several sequence features can be assigned to anchor TMHs: charged-residue flank biases, leucine intra-helix asymmetry and the “aromatic belt”

We investigated the difference between TMHs from single-pass and multi-pass proteins and found significant differences in sequence composition that are reflective of the biologically different roles the TMHs play. To emphasise and validate these findings, we separated TMHs from single-pass proteins into simple and complex TMHs [6, 7]: one type that likely contains mostly TMHs that act as exclusive anchors, and another that has roles beyond anchorage. This leaves us with “anchors” (simple TMHs from single-pass proteins) and “non-anchors” (complex TMHs from single-pass proteins and TMHs from multi-pass proteins). If there are strong sequence feature differences between anchors and non-anchors, it is likely that the sequence feature has a role in satisfying membrane constraints to act as an energetically optimally stable anchor.

Future studies in the area would desirably directly include a comprehensive analysis of datasets of oligomerised TMHs from single-pass proteins and ascertain if they appear to be more similar to simple anchors, multi-pass proteins or generally neither. Currently, no sufficiently complete set of intra-membrane oligomerised single-pass proteins exists that can be compared to a large set of known non-oligomerising proteins. The current work sidesteps this issue by comparing single-pass proteins with simple TMHs, which tend to be simple anchors (as shown in previous work [6, 7]), against datasets that contain TMHs that will form intra-membrane bundles. Bluntly, the simple/complex status of a TMH can be easily computed from its sequence with TMSOC, whereas the oligomerisation state of most membrane proteins still needs to be experimentally determined.

Unsurprisingly, both positively and negatively charged residues can be seen to be more strongly distributed with bias in anchors than non-anchors. Both the “positive-inside” rule and the “negative-not-inside/negative-outside” bias are mostly observable in simple single-pass TMHs (although they are statistically significant elsewhere). It is perhaps true that where a bias is clearly present in both non-anchors and anchors alike, it is a strong topological determinant, whereas if the residue is only distributed with topological bias in exclusively anchoring TMHs, we can attribute these features more specifically to biophysical anchorage. This being said, we should not rule out that the same features aid topological determination, since negative charge has been shown to be a weaker topological determinant than positively charged residues [35].

Tyrosine and tryptophan residues commonly are found at the interfacial boundaries of the TMH, and this feature, called the "aromatic belt'' [9, 13, 14, 31, 36], was thought to be caused by their affinity to the carbonyl groups in the lipid bilayer [77]. Not all types of aromatic residues are found in the aromatic belt; phenylalanine has no particular preference for this region [14, 78]. It is still unclear if the aromatic belt has to do with anchorage or with translocon recognition [13]. Here, TMHs with exclusively anchorage functions showed stronger preferences for the W and Y in the aromatic belt region, otherwise known as the water-lipid interface region, than TMHs with function beyond anchorage. This is strong evidence that the aromatic belt indeed assists with anchorage and is less conserved where the TMH must conform to other restraints beyond membrane anchorage. Furthermore, we see that tyrosine's preference for the inside interface region also appears to be involved with anchorage, and this trend is somewhat true for tryptophan, too.

Finally, our findings corroborate earlier reports that many multi-pass TMHs are much less hydrophobic than typical single-pass TMHs and about 30% of them fail the hydrophobicity requirements of ΔG TMH insertion prediction (“missing hydrophobicity”) [36, 63–65]. We also find that the leucine skew and the hydrophobic asymmetry towards the cytosolic leaflet of the membrane are more pronounced in simple, single-pass TMHs than in complex or multi-pass ones; thus, they appear to be another anchoring feature. It was found previously that the hydrophobic profiles of TMHs of multi-pass proteins share similar hydrophobicity profiles on average irrespective of the number of TMHs, and TMHs from single-pass proteins have been found to be typically more hydrophobic than TMHs from multi-pass proteins [6]. Sharpe et al. [9] report an asymmetric hydrophobic length for single-pass TMHs. Our study reiterates the hydrophobic asymmetry and attributes it mainly to the leucine distribution. The leucine asymmetry might be linked to the different lipid compositions of either leaflet of biological membranes.

## Conclusion

**Fig. 8:**
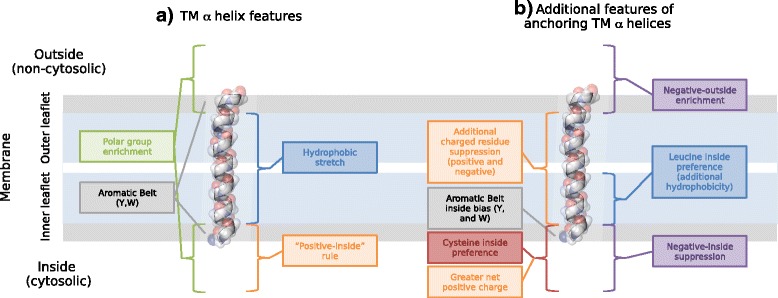
Residue distributions of transmembrane anchors. A view showing additional residue distribution features that TMHs with an anchorage function display. **a** The more classic model of a TMH showing the "positive-inside" rule [12], the hydrophobic core [52], the polar enrichment that flanks the hydrophobic stretch [13] and the aromatic belt [14]. **b** Simple anchors may display additional features that conform to the membrane biophysical constraints: further suppression of charge in the hydrophobic core (Table 1), intra-membrane leucine asymmetry that likely causes hydrophobic skew [9] (Table 4, Fig. 5), a higher preference for cysteine on the inside flanking region (Fig. 7k and l), a higher net "positive-inside" charge (Additional file 1: Figure S1), asymmetric skew of the hydrophobic belt favouring the inner leaflet interface (Fig. 7e, f, g and h) and a negative-outside bias via suppression on the inside flanking region or enrichment on the outside flanking region (Fig. 7c and d, Tables 2 and 3)

 In summary, three key features can be assigned that aid TMH stability in the membrane (Fig. 8): (1) charge, (2) the aromatic belt, and (3) leucine leaflet preference. What is most novel here is that each of these features is furthermore distributed with preference for a particular side of the bilayer in the case of anchoring TMHs. These differences in inside-outside topology that are most present in anchoring TMHs further support the notion that there are broad lipid compositional differences between the inner and outer leaflets of the bilayers [9]. Furthermore, whilst some TMHs conform and complement to the properties of the bilayer, other TMHs with function beyond anchorage are less constrained to biophysically complement the bilayer. For these TMHs, any advantage gained by adhering to the membrane restrictions is outweighed by more complicated protein dynamics, topological frustration and protein functional requirements.

To conclude, the large fraction of functionally uncharacterised genomic sequences is the great bottleneck in life sciences at this moment that hinders many biomedical and biotechnological applications, some with tremendous societal need [27, 79]. Amongst these uncharacterised genomic regions, there are ~10,000 protein-coding genes, especially many membrane-embedded proteins. It is hoped that the NNI/NO rule as well as the other sequence properties of membrane anchoring TMHs described in this article will add new insights for membrane protein function discovery, design and engineering.

## Methods

### Datasets

All datasets used for analysis are listed in Table 1. Transmembrane protein sequences and annotations were taken from TOPDB [50] and UniProt [49]. UniProt-derived datasets are the most comprehensive datasets, built with (1) robust transmembrane prediction methods, providing the limit of today’s achievable accuracy with regard to hydrophobic core localisation, and (2) subcellular location annotation that can be used for orientation determination. However, they mostly rely on predicted transmembrane regions. TOPDB has meticulous experimental verifications of the orientation from the literature that are independent of prediction algorithms [50]. Unfortunately, this dataset is much smaller with too few entries to have it divided with regard to taxonomy or subcellular locations.

UniProt database files were downloaded by querying the server for different taxonomic groups as well as different subcellular membrane locations: UniHuman (human representative proteome), UniCress (*Arabidopsis thaliana*, otherwise known as mouse-ear cress, representative proteome), UniER (human endoplasmic reticulum representative proteome), UniPM (human plasma membrane representative proteome) and UniGolgi (human Golgi representative proteome). To enforce a level of quality control, the queries were restricted to manually reviewed records and transmembrane proteins with manually asserted TRANSMEM annotation [49]. Proteins were then sorted into multi-pass and single-pass groups according to whether they had more than one or exactly one TRANSMEM region respectively. TRANSMEM regions are validated by either experimental evidence [49] or according to a robust transmembrane consensus of the predictors TMHMM [23], Memsat [80], Phobius [21, 22] and the hydrophobic moment plot method of Eisenberg and co-workers [54]. TMHs and flanking regions were oriented according to UniProt TOPO_DOM annotation according to the keyword “cytoplasmic”. If a “cytoplasmic” TOPO_DOM was found in the previous TOPO_DOM relative to the TRANSMEM region, then the sequence remained the same. If “cytoplasmic” was found in the next TOPO_DOM, relative to the TRANSMEM section, then the sequence was reversed. Proteins without the “cytoplasmic” keyword in their TOPO_DOM annotation were omitted from further analysis.

The TOPDB database [50] is a manually curated database composed of experimental records from the literature that allow determination of the protein topology. Experiments include fusion proteins, posttranslational modifications, protease experiments, immunolocalisation, chemical modifications as well as revertants, sequence motifs with known mandatory membrane-embedded topologies and tailoring mutants (Additional file 3: Table S1). Length cut-offs for the TMH were set with 16 as the shortest length and 38 as the longest.

The datasets described in the following subsections are used throughout this work.

#### ExpAll

TOPDB contained 4190 manually annotated transmembrane proteins at the time of download [50]. CD-HIT [81] identified 3857 representative sequences using sequence clusters of >90% sequence identity. This choice of similarity threshold was chosen since CD-HIT ultimately underlies the clustering behind UniRef. Unlike the other datasets, which by definition contain reasonably typical TMHs, many of the transmembrane segments annotated in TOPDB are extremely short or long, and this would cause severe unrealistic hydrophobic mismatches. The short segments in particular could be the result of misannotation, TMHs broken into pieces due to kinks or segments that peripherally insert only into the interface of the membrane bilayer. To remove the atypical lengths, cut-offs were set with 16 as the lower cut-off and 38 as the upper cut-off after inspecting the length histogram. We found that, for the single-pass TMHs in TOPDB, 1215 out of 1544 are within the length limits (78.7%). Amongst the 17,141 multi-pass TMHs, we find 15,563 within our global length limits (from 2205 TOPDB records corresponding to 2281 UniProt entries). This removed 1578 very short TMHs and none of the long TMHs. Our cut-off selection is very similar to the one used by Baeza-Delgado et al. [13].

To get an idea of the taxonomical breakdown in the ExpAll dataset, the UniProt ID tags were extracted and mapped to UniProtKB. The combined dataset of multi-pass (single-pass) proteins was mapped to 1288 (1343) eukaryotic records, 404 (776) of which were human records, 926 (191) bacterial records, 46 (5) Archaea records and 14 (22) viral records.

#### UniHuman

This is a set of mostly human TMH-containing proteins or their close mammalian homologues. UniProtKB contains 5187 human protein records that are manually annotated with TRANSMEM regions (query = “annotation:(type:transmem) AND reviewed:yes AND organism:"Homo sapiens (Human) [9606]" AND proteome:up000005640”. To reduce sequence redundancy, these sequences were submitted to UniRef90 [82]. To note, UniRef90 was chosen over UniRef50 to maintain a viable size of datasets for statistical analysis of occurrence of negatively charged residues, which are very rare in the vicinity of TMHs. There were 5015 UniRef90 clusters representing the 5187 sequences. A list of sequences representing those clusters was submitted back to UniProtKB, and 5014 representative entries were recovered. There is a small issue in that the list of representatives from UniRef includes non-canonical isoforms, whilst the batch retrieve query of UniProtKB only supports complete entries, i.e. canonical isoforms. This resulted in the loss of one record at this point due to two splice isoforms acting as representative identifiers. Of those 5014 records, 4714 were records from human entries, 197 were from mice, 94 from rats, 5 from bovines, 2 from chimps, 1 from Chinese hamsters, and 1 from pigs. Although the TMH length variations within the UniHuman dataset are much smaller than for ExpAll, we applied the same length cut-offs for the sake of comparability. Out of the 1709 single-pass cases, 1705 entered the final dataset. Of those, 1596 were from human records, 87 were from mouse, 19 were from rat, and 2 were from chimpanzee. The further loss of a record in the taxonomic query is again due to multiple splice isoform records being represented by a single UniProt record. Amongst the 12,390 multi-pass TMHs, 12,353 were included into UniHuman. The other, multi-pass record identifiers were mapped to 1789 UniProtKB entries. Of these, 1660 were human entries, 63 from rat, 61 from mouse, 4 from bovines and 1 from Chinese hamsters. This clustered human dataset was then queried for subcellular locations to make the UniER, UniGolgi and UniPM datasets (detailed below).

#### UniER

The clustered UniHuman dataset was queried using UniProtKB for endoplasmic reticulum subcellular location (locations:(location:"Endoplasmic reticulum [SL-0095]" evidence:manual)). This returned 487 protein entries, 457 of which belonged to human, 24 to mouse and 6 to rat. Of these records, 287 contained sufficient annotation for orientation determination. One hundred thirty-two were single-pass entries, of which 120 records were from humans, 11 from mouse, and 1 from rat. One hundred fifty-five were multi-pass entries containing 898 TMHs. One hundred forty-four were records from human, 8 were from mouse and 3 were from rat.

#### UniGolgi

The clustered human dataset was queried using UniProtKB for Golgi subcellular location (locations:(location:"Golgi apparatus [SL-0132]" evidence:manual)). This returned 323 protein entries, 301 of which belonged to human, 19 to mice, 2 to rat and 1 to pig. Of these records, 269 contained sufficient annotation for orientation determination. Two hundred six were single-pass entries, of which 195 records were from human, 9 from mouse, and 1 from rat. Sixty-one were multi-pass entries containing 383 transmembrane regions. Fifty-four were records from human, 6 were from mouse and 1 was from rat.

#### UniPM

The clustered human dataset was queried using UniProtKB for the cell membrane subcellular location (locations:(location:"Cell membrane [SL-0039]" evidence:manual)). This returned 1036 protein entries, 948 of which belonged to humans, 62 to mice, and 26 to rats. Of these records, 920 contained sufficient annotation for orientation determination. Four hundred ninety-three were single-pass entries, of which 451 records were from human, 37 from mouse, and 5 from rat. Four hundred twenty-seven were multi-pass entries containing 3079 transmembrane regions. Three hundred ninety-four were records from human, 17 were from mouse and 16 were from rat.

#### UniCress

For the mouse-ear cress, a representative proteome dataset was acquired with the query annotation:proteomes:(reference:yes) AND reviewed:yes AND organism:"Arabidopsis thaliana (Mouse-ear cress) [3702]" AND proteome:up000006548. This returned 3174 records in UniProtKB. UniRef90 identified 3111 clusters. Of the representative sequences, 3110 were mapped back to UniProtKB. Of those, 3090 were from *Arabidopsis thaliana*, 2 from Hornwort, 1 from cucumber, 1 from tall dodder, 1 from soybean (*Glycine max*), 2 from Indian wild rice, 2 from rice, 2 from garden pea, 1 from potato, 4 from spinach, 1 from *Thermosynechococcus elongatus* (thermophilic cyanobacterium), 1 from wheat, and 2 from maize. Of those there were 1146 with suitable TOPO_DOM annotation for topological orientation determination. Of those records, 632 were identified as single-pass, all of which were from *Arabidopsis thaliana*. Five hundred seven protein records were from multi-pass records, which contained 3823 TMHs. Five hundred six of those records were from *Arabidopsis thaliana,* whilst 1 was from *Thermosynechococcus elongatus*.

#### UniFungi

For the Fungi dataset, the query “annotation:(type:transmem) taxonomy:"Fungi [4751]" AND reviewed:yes” was used. This returned 5628 records that were submitted to UniRef90. UniRef90 identified 4934 representative records, all of which were successfully mapped back to UniProtKB. Of those, 2070 had suitable annotation for orientation. A total of 1990 records belonged to Ascomycota including 1243 Saccharomycetales. 73 were Basidiomycota, and 6 were Apansporoblastina. Seven hundred twenty-nine records contained a single TMH region, 702 of which belonged to Ascomycota, 26 to Basidiomycota and 1 to *Encephalitozoon cuniculi*, a Microsporidium parasite. There were 8698 helices contained in 1338 records of multi-pass proteins. Of these records, 1285 were Ascomycota, 47 were Basidiomycota, and 5 were Apansporoblastina. One TMH from UniFungi was discounted from P32897 due to an unknown position.

#### UniEcoli

This dataset was generated by querying UniProt with “reviewed:yes AND organism:”Escherichia coli (strain K12)[83333]””, which returned 941 hits. The hits were submitted to UniRef90, which returned 935 clusters. The representative IDs were then resubmitted to UniProtKB, all of which returned successfully. Nine hundred thirty-four were from bacteria, whilst one was from lambdalike viruses. Of the bacterial records, 862 were from various *Escherichia* species, of which 565 were from *E. coli* strain K12, 28 were from *Salmonella choleraesuis*, 25 were from *Shigella* and the rest all also fell under the Gammaproteobacteria class. This dataset contains 54 single-pass proteins and 3888 helices from 529 multi-pass proteins with sufficient annotation for topological determination.

#### UniBacilli

The Bacilli dataset was constructed by querying UniProt for “reviewed:yes AND taxonomy:”Bacilli””. This returned 5044 records, which were submitted to UniRef90. There were 2591 clusters found in UniRef from these records. The representative IDs were successfully resubmitted to UniProtKB. Of these, 2031 were of the order Bacillales whilst 560 were also of the order Lactobacillales. This dataset contains 124 single-pass proteins and 822 helices from 140 multi-pass proteins.

#### UniArch

The Archaea dataset was constructed by querying UniProt for “reviewed:yes AND taxonomy:”Archaea [2157]””. This returned 1152 records, which were submitted to UniRef90. One thousand fifty-four clusters were found in UniRef from these records. The representative IDs were successfully resubmitted to UniProtKB. Nine hundred forty-six records belonged to the Euyarchaeota, 101 to Thermoprotei, 4 to Thaumarchaeota, and 3 to *Korarchaeum cryptofilum*. This dataset contains 48 single-pass proteins and 59 multi-pass proteins containing 327 helices from 59 proteins.

We are aware that proteome datasets are “moving targets” that have dramatically changed over the years and probably will continue to do so to some extent in the future [83]. Yet, we think that currently available protein sequence sets are sufficiently good for our purposes, as we search for statistical properties in the TMH context only.

### On the determination of flanking regions for TMHs and the TMH alignment

The determination of the boundary point at the sequence between the TMH in a membrane and the sequence immersed in the cytoplasm, extracellular space, vesicular lumen, etc. is not as trivial as it initially appears. There is a lot of dynamics in the TMH positioning, and the actual boundary point will be represented by various residues at different time points. Whilst the TMH core region detection from a sequence is trivial with modern software, the exact determination of TMH boundaries remains difficult, since it is unclear exactly how far in or out of the membrane a given helix extends [84]. Previous studies have dealt with this issue in various ways [9, 13, 16, 85].

Here in this work, we explore two boundary definitions. First, we assign TMH boundary locations as described in the respective databases. These flanks are the ones that are reported in our TMH data files that are available at http://mendel.bii.a-star.edu.sg/SEQUENCES/NNI/. We studied flank lengths of ±5, ±10, and ±20 residues preceding and following the inside and outside TMH boundaries. In these cases, the flanks are aligned relative to the residue closest to the TMH.

In cases where the loops before and after the TMH are shorter than the predefined flank lengths, further precautions are necessary. In the multi-pass datasets particularly (Additional file 4: Figure S4, Additional file 3: Table S1), the flanks overlap with other membrane region flanks. We explore several variants. On the one hand, we work with data files where the flank residue stretches are equally truncated so that no overlap occurs. If the loop length was uneven, the central odd residue was not included into any flank. We find, surprisingly, that a large number of TMHs have no or just a super-short flank, a circumstance that should disturb any statistical analysis due to the absence of objects. Therefore, we also work with alternative datasets: (1) with flanks overlapping between consecutive TMHs (e.g. in Table 3B, yet this leads to some residues being counted more than one time) as well as (2) with subsets of the data where the flanks at both sides have a defined minimal length (50% or 100% of the required flanks; unfortunately, some of them become too small for analysis).

The problem of flanks overlapping also affects some single-pass and multi-pass TMH proteins with INTRAMEM regions as described in some UniProt entries. We do not include INTRAMEM regions in the datasets as TMHs, but sometimes the flanking regions of TMHs were truncated to avoid overlap with INTRAMEM flanking regions (Additional file 5: Table S2). The identifiers affected for single-pass TMH proteins are Q01628, P13164, Q01629, Q5JRA8, A2ANU3 (UniHuman), P13164, Q01629, A2ANU3 (UniPM) and Q5JRA8 (UniER).

The second form of boundary point definition for flank determination was achieved by gaplessly aligning all TMHs relative to their central residue at the position equal to half the length of the TMHs at either side. Though there is some length variation amongst TMHs; most of them are centred around a length of 20–22 residues. In this case, flanks are the sequence extensions beyond the standardised-length 21-residue TMHs. We define the inside flanking segments as the positions –20 to –10 and the outside flanking regions to be +10 to +20 from the central TMH residue (with the label “0”). Instead of emphasising some artificially selected boundary residue, this definition allows the average TMH boundary transition to become apparent.

### Separating simple and complex single-pass helices

Single-pass helices from ExpAll and UniHuman datasets helices were split into two groups: simple and complex following a previously described classification [6, 7] to roughly distinguish simple hydrophobic anchors and TMHs with additional structural/functional roles. Simple and complex helices were determined using TMSOC [7]. The complexity class is determined by calculating the hydrophobicity and sequence entropy. The resulting coordinates cluster with anchors being more hydrophobic and less complex, whilst more complex and more polar TMHs are associated with non-anchorage functions. In UniHuman there were 889 simple helices and 570 complex TMHs. In ExpAll there were 769 simple helices and 570 complex helices.

### Distribution normalisation

In this work, we have used normalisation techniques described in previous investigations as well as new approaches designed to more sensitively identify biases of rare residues. Baeza-Delgado and co-workers used LogOdds normalisation column-wise in TMH alignments. Critically, this is based on their definition of probability, which takes into account the total number of amino acids in the dataset as a denominator [13]. Since aliphatic residues such as leucine and other highly abundant slightly polar residues dominate the denominator, the distribution of the rare acidic residues will be easily lost in the “background noise” of those highly abundant residues. Pogozheva and co-workers used two approaches, (1) the total accessible surface area (*ASA*_total_) and (2) the total number of charged residues (*N*_total_), as a denominator in their distribution normalisation [16].

In this work, two methods for measuring residue occurrence in the TMH and its flanks were used. As in previous work, we compute the occurrence *a*_*i*,*r*_ of an amino acid type *i* at a certain sequence position *r* in a set of aligned sequences of TMHs and their flanks. Following [9], the absolute relative occurrence *p*_*i*,*r*_ of this amino acid type at the sequence position *r* is then given by Eq. (1) as:

1 $$ {p}_{i,r}=\frac{a_{i,r}}{\underset{r}{max}\left({a}_r\right)} $$

Here, the denominator is the maximal number of all residues in any alignment column (i.e., the number of sequences in the alignment) and, to emphasise, this will make *p*_*i*,*r*_ mostly dependent on the most abundant residue types. This type of normalisation reveals the most preferred residue types at given sequence positions.

Our second normalisation method is independent of the abundance of any amino acid types other than the studied one, and it answers the question: If there is a residue of type *i* in the TMH-containing segment, where would it most likely be? This relative occurrence *q*_*i*,*r*_ is calculated in Eq. (2) as:

2 $$ {q}_{i,r}=\frac{100\cdot {a}_{i,r}}{a_i} $$

The value *a*_*i*_ is the total abundance of residues of just amino acid type *i* in a given alignment of TMH-containing segments (i.e., in the TMH together with its two adjoining flanks summed over all cases of TMHs in the given dataset). Peaks in *q*_*i*,*r*_ as a function of *r* reveal the preferred positions of residues of type *i*. The difference in *p*_*i*,*r*_ and *q*_*i*,*r*_ normalisation is visualised in Additional file 6: Figure S3.

### Hydrophobicity calculations

Hydrophobicity profiles were calculated using the Kyte and Doolittle hydrophobicity scale [52] and validated with the Eisenberg scale [54], the Hessa biological scale [36] and the White and Wimley whole residue scale [53] (Additional file 1: Figure S1). The hydrophobicity profile uses un-weighted windowing of the residue hydrophobicity scores from end to end of the TMD slice. Three residues were used as full window lengths, and partial windows were permitted.

### Normalised net charge calculations

Charge was calculated at each position by scanning through each position of the TMHs and flanking regions and subtracting one from the position if an acidic residue (D or E) was present, or adding one if a positively charged residue (K or R) was present. The accumulative net charge *c*_*r*_ was then divided by the total number *N* of TMHs that were used in calculating the accumulative net charge. Thus, the charge distribution is calculated by:

3 $$ {c}_r=\frac{\left({a}_{K,r}+{a}_{R,r}\right)-\left({a}_{D,r}+{a}_{E.r}\right)}{N} $$

### Statistics

The inside/outside bias of negative residues was quantified by computing the independent Kruskal-Wallis (KW) and two-sample *t* test statistical method from the Python scipy.stats package v0.15 (https://docs.scipy.org/doc/scipy/reference/generated/scipy.stats.kruskal.html, https://docs.scipy.org/doc/scipy-0.15.0/reference/generated/scipy.stats.ttest_ind.html). This test answers the question of whether two means are actually different in the statistical sense. For the leucine residues, each TMH region was divided into two sections, representing the inner and outer leaflets (Table 4). For the hydrophobicity plot, three window values of hydrophobicity were taken for each TMH at each position. The statistical analyses were separately performed for single-pass and multi-pass transmembrane proteins. At each position, the two groups were compared using the KW test.

The zero hypothesis of homogeneity of two distributions was examined with the Kolmogorov-Smirnov (KS), the KW and the χ^2^ statistical tests.The KS test scrutinises for significant maximal absolute differences between distribution curves, the KW test looks for skews between distributions and the χ^2^ statistical test checks the average difference between distributions. As the statistical significance value (*P* value) is a strong function of *N*, the total amount of data used in the statistical test, we rely on the (absolute) Bahadur slope (*B*) as a measure of distance between two distributions [55–57]:

4 $$ B=\frac{\left| ln\left(P- value\right)\right|}{N} $$

The larger the absolute Bahadur slope, the greater the difference between the two distributions.

## Additional files

Additional file 1: Figure S1. The net charge per TMH plotted at each position; the positive-inside rule is stronger in TMHs from single-pass proteins than TMHs from multi-pass proteins. The net charge was calculated at each position as described in the Methods section for the (A) UniHuman and (B) ExpAll datasets. Net charge for TMHs from multi-pass proteins is shown in *black*, and the profile of TMHs from single-pass proteins is drawn in *blue*. (PDF 17 kb)

Additional file 2: Figure S2. The difference in hydrophobicity between the single-pass and multi-pass datasets stratified by number of TMHs is not due to the choice of scale. As with Fig. 5, UniHuman was stratified according to the number of TMHs in each protein. The mean amino acid hydrophobicity values of TMHs with a sliding unweighted window of 3 residues from UniHuman proteins at each position were plotted. To validate the findings presented in Fig. 5a, several scales of hydrophobicity were used. (A) The White and Wimley whole residue scale [53] is based on the partitioning of peptides between water and octanol as well as water to POPC. A positive score indicates a more polar score. (B) The Hessa biological scale [36]. The hydrophobicity values represent the free energy exchange during recognition of designed peptide TMHs by the endoplasmic reticulum Sec61 translocon and, therefore, negative values indicate an energetic preference for the interior of a lipid bilayer. (C) Eisenberg’s consensus scale [54] is a scale based on the earlier scales from Nozaki and Tanford [86], Wolfenden et al. [87], Chothia [88], Janin [89] and the von Heijne and Blomberg scale [90]. The scales are normalised according to serine. A positive score indicates a generally more hydrophobic score. (PDF 43 kb)

Additional file 3: Table S1. The experimental evidences of TOPDB. The total number of experimental evidences that contribute to ExpAll according to the TOPDB database (more information is available at http://topdb.enzim.hu/?m=exptype&mid=14). * refers to the total number of a subsection being larger than the total of the subcategories, likely due to lack of annotation where ambiguous literature evidence is counted towards the total but cannot be categorised further. (DOC 47 kb)

Additional file 4: Figure S4. The lengths of flanks and TMHs in multi-pass and single-pass proteins in the UniHuman and ExpAll dataset. On the *horizontal axis* are the lengths of the TM segment regions in residues. On the *vertical axis* are the percentages of the population. There are three regions: the inside flank, the TMH and the outside flank. These regions are acquired according to the TMH boundary of the respective database. Where no overlap is permitted, if the flank encroaches the flank of another TMH, the flank length becomes half the number of residues in the loop region between the two features. Where they are allowed to overlap, flanking residues may include other flanks, or indeed other TMHs. (PDF 410 kb)

Additional file 5: Table S2. Records with INTRAMEM and TRANSMEM flanking region overlap. The total number of TMHs from UniProt datasets with flanking region overlap between INTRAMEM and TRANSMEM regions. The number of multi-pass records to which the TMHs belong are shown in brackets. (DOC 39 kb)

Additional file 6: Figure S3. Relative percentage heatmaps from the predictive datasets calculated by fractions of the absolute maximum and by the relative percentage of a given amino acid type. The residue position aligned to the centre of the TMH is on the *horizontal axis*, and the residue type is on the *vertical axis*. Amino acid types are listed in order of decreasing hydrophobicity according to the Kyte and Doolittle scale [52]. The flank lengths in the TMH segments were restricted to up to ±5 residues. The scales for each heatmap are shown beneath the respective subfigure. All TMHs and flank lengths are from the UniHuman dataset. (A) The heatmap has been coloured according to a scale that uses column-wise normalisations used in previous studies [9]. See Eq. (1) in the Methods section. As an illustrative example, we show how the value for E at position ±12 is obtained. There are in total 91/22 Es at these positions in 1705 sequences; thus, the represented value is 0.013 at –12 and 0.053 at 12. Note that L is clearly a hotspot as well as trends for other hydrophobic residues, I and V, as is to be expected. A positive inside effect can also be seen. (B) The heatmap has been coloured according to the relative percentage of each amino acid type. Here, 91/22 Es at position ±12 are compared with 615 Es seen within the flanks and the TMH section itself amongst all sequences in the alignment. So, the expectation of an E at position ±12 if there is any E in the TMH + flanks region at all is 0.036 at –12 and 0.148 at position 12. With this type of normalisation, not surprisingly, we see the positive-inside rule is hotter than in subfigure A. There are also hotspots in the flanks for the negatively charged residues on the outside flank. The leucine hotspot is no longer very pronounced, as the leucines are quite evenly spread over many positions. (PDF 120 kb)
