# Erratum to: Charged residues next to transmembrane regions revisited: “Positive-inside rule” is complemented by the “negative inside depletion/outside enrichment rule”

**DOI:** 10.1186/s12915-017-0410-6

**Published:** 2017-08-18

**Authors:** James Alexander Baker, Wing-Cheong Wong, Birgit Eisenhaber, Jim Warwicker, Frank Eisenhaber

**Affiliations:** 1 Bioinformatics Institute, Agency for Science Technology and Research (A*STAR), 30 Biopolis Street #07-01, Matrix, Singapore, 138671 Singapore; 2 School of Chemistry, Manchester Institute of Biotechnology, 131 Princess Street, Manchester, M1 7DN UK; 3 School of Computer Engineering (SCE), Nanyang Technological University (NTU), 50 Nanyang Drive, Singapore, 637553 Singapore

## Erratum

Upon publication of the original article [1], the authors noticed that an error was introduced at proofing stage in the panel labels of Fig. 1. Instead of reading ‘a; b; c; d’, these read ‘a; b; c; c’. This has now been updated in the original article and the correct version is included in this erratum. Inconsistencies in the use of brackets around the panel labels of the figure legends have also been corrected and each panel label is now displayed in brackets. We apologise for any inconvenience caused by these errors.

Please see below the correct Fig. 1:

**Fig. 1:**
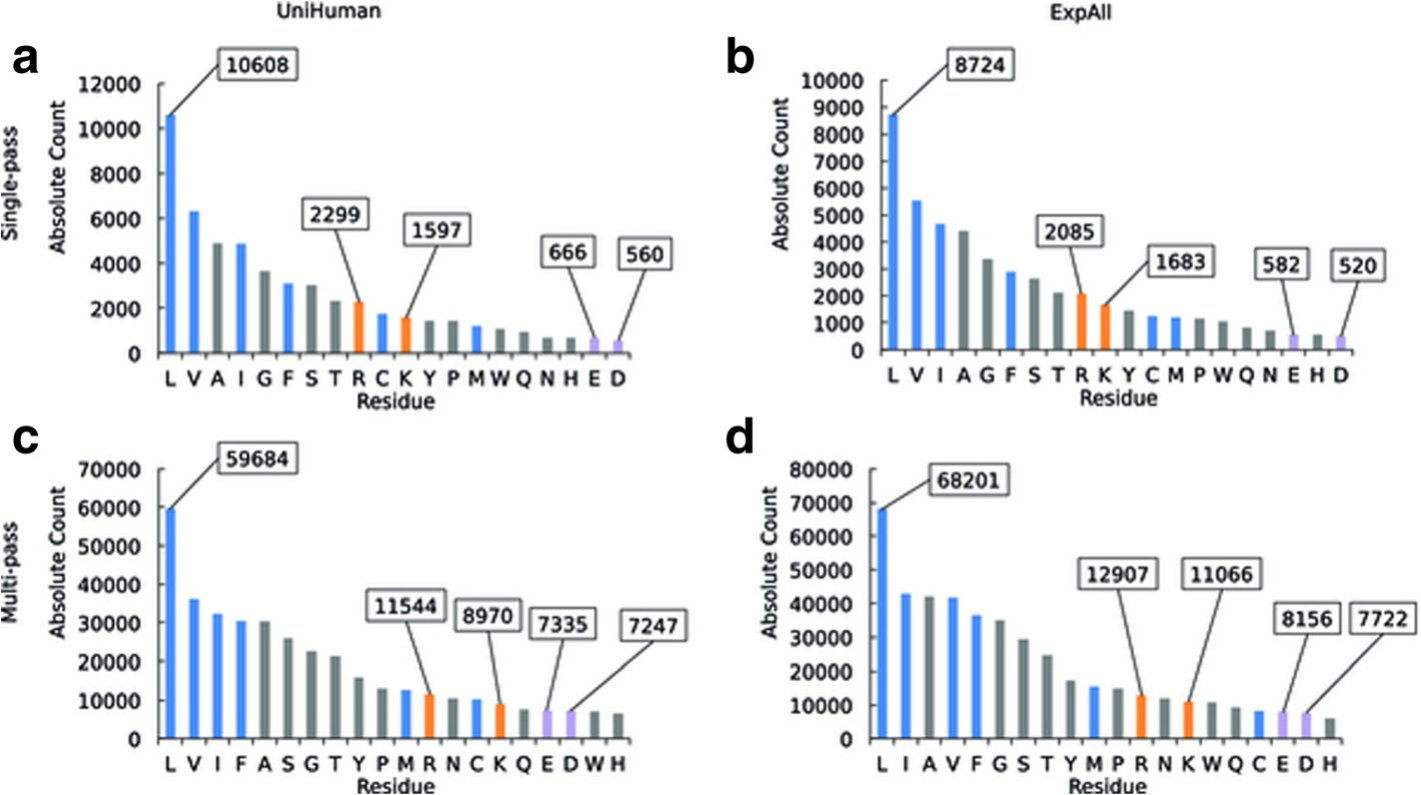
Negatively charged amino acids are amongst the rarest residues in TMHs and ±5 flanking residues. Bar charts of the abundance of each amino acid type in the TMHs with flank lengths of the accompanying ±5 residues from the (**a**) UniHuman single-pass proteins, (**b**) ExpAll single-pass proteins, (**c**) UniHuman multi-pass proteins, and (**d**) ExpAll multi-pass proteins. Amino acid types on the *horizontal axis* are listed in descending count. The bars were coloured according to categorisations of hydrophobic, neutral and hydrophilic types according to the free energy of insertion biological scale [36]. *Grey* represents hydrophilic amino acids that were found to have a positive ΔGapp, and *blue* represents hydrophobic residues with a negative ΔGapp, *purple* denotes negative residues and positive residues are coloured in *orange*. The abundances of key residues are labelled
