# Supplementary figures and images for: Charged residues next to transmembrane regions revisited: “Positive-inside rule” is complemented by the “negative inside depletion/outside enrichment rule”
Source: BMC Biol. 2017 Jul 24;15:66. doi: 10.1186/s12915-017-0404-4 (PMC5525207; doi:10.1186/s12915-017-0404-4)

A) UniHuman

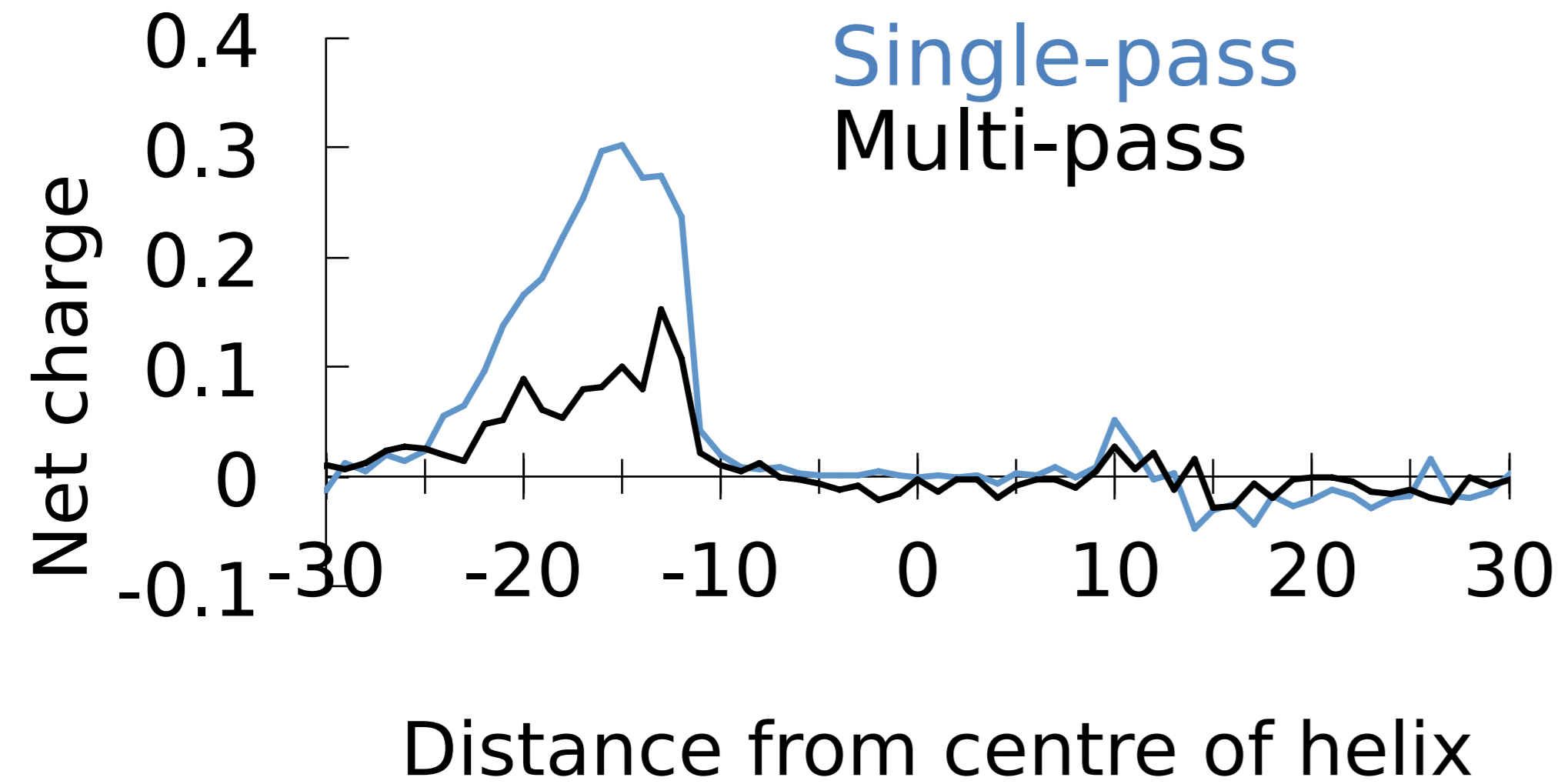

B) ExpAll

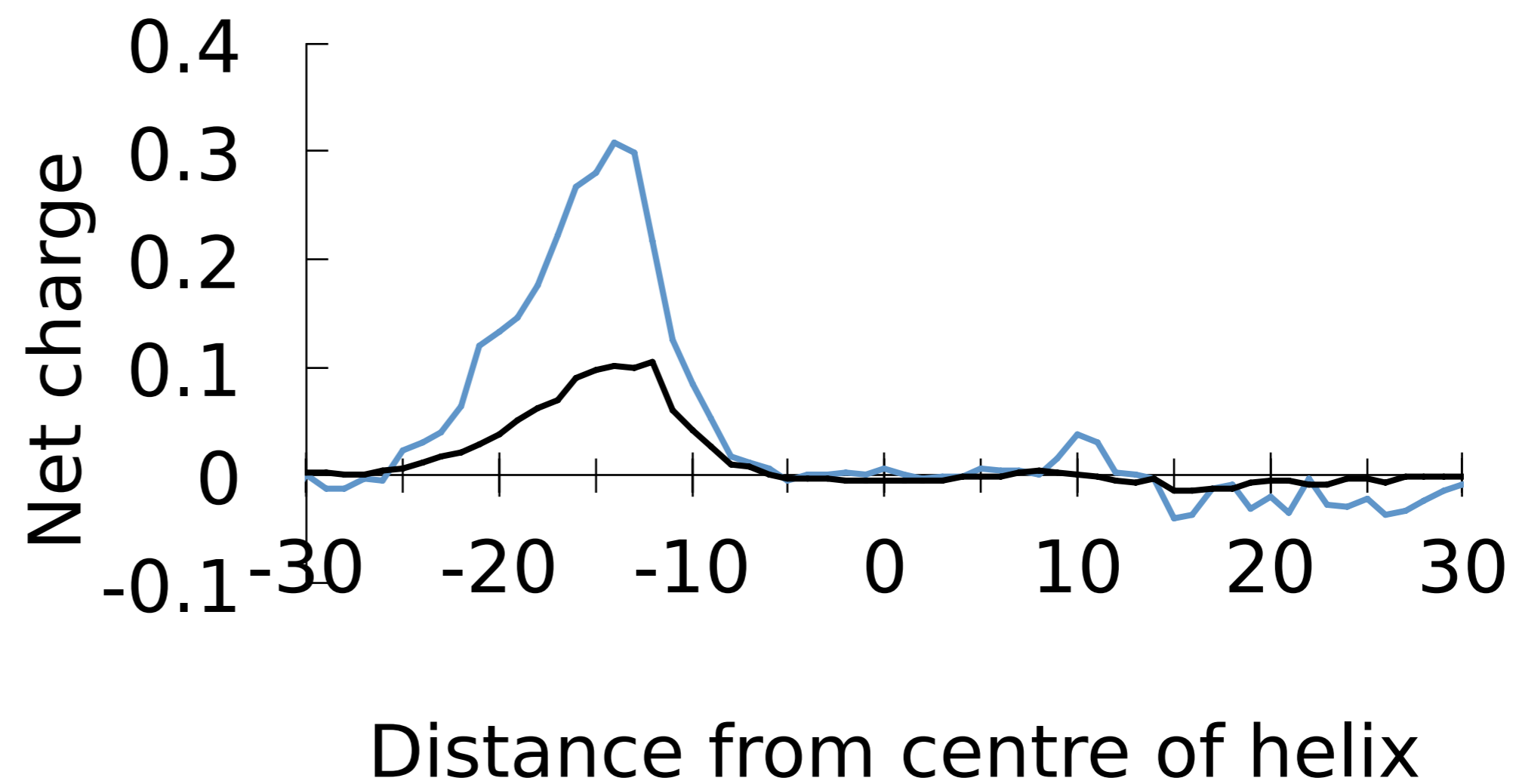

Supplement: Supplementary file 1 — The net charge per TMH plotted at each position; the positive-inside rule is stronger in TMHs from single-pass proteins than TMHs from multi-pass proteins. The net charge was calculated at each position as described in the Methods section for the (A) UniHuman and (B) ExpAll datasets. Net charge for TMHs from multi-pass proteins is shown in black, and the profile of TMHs from single-pass proteins is drawn in blue. (PDF 17 kb) [file 12915_2017_404_MOESM1_ESM.pdf]

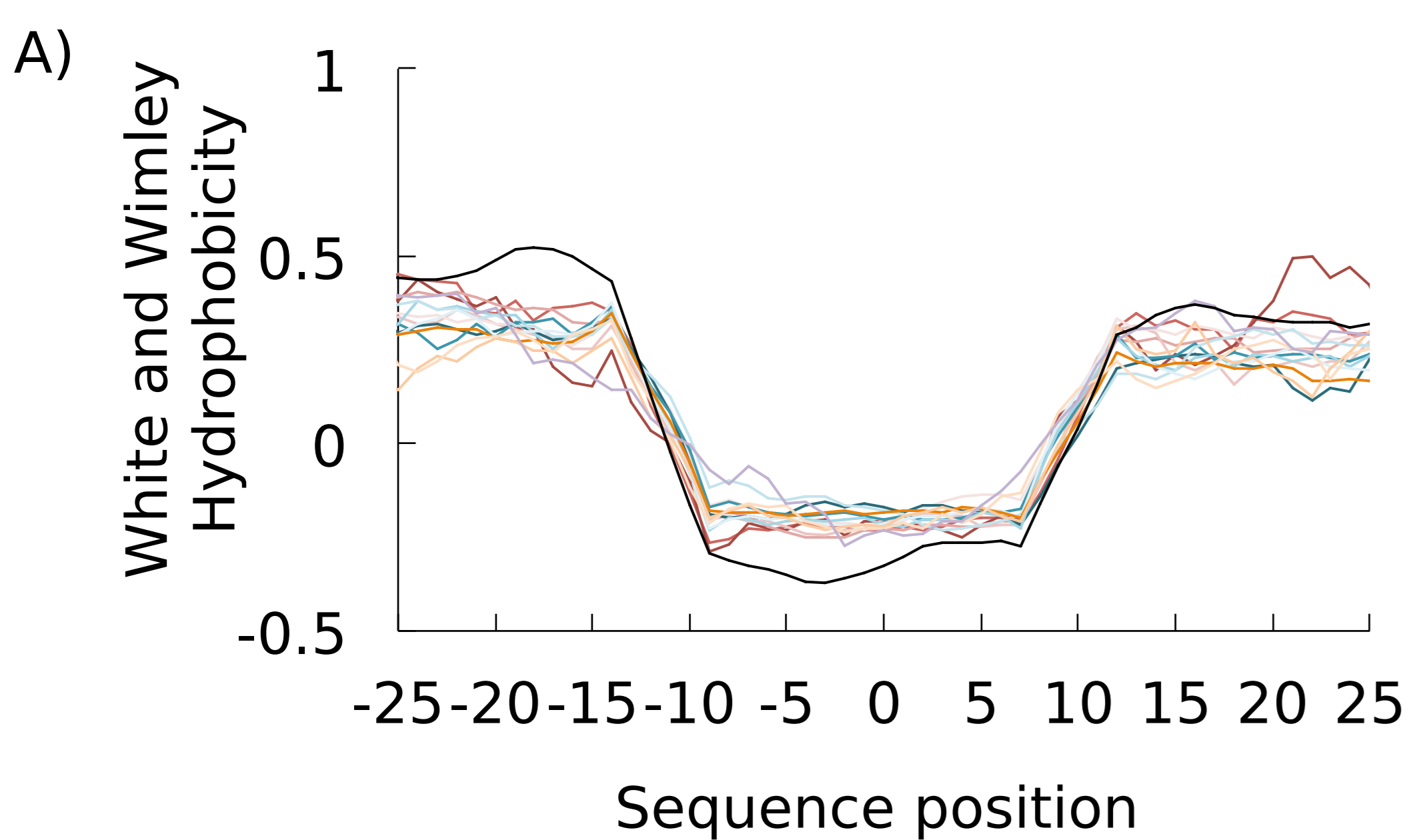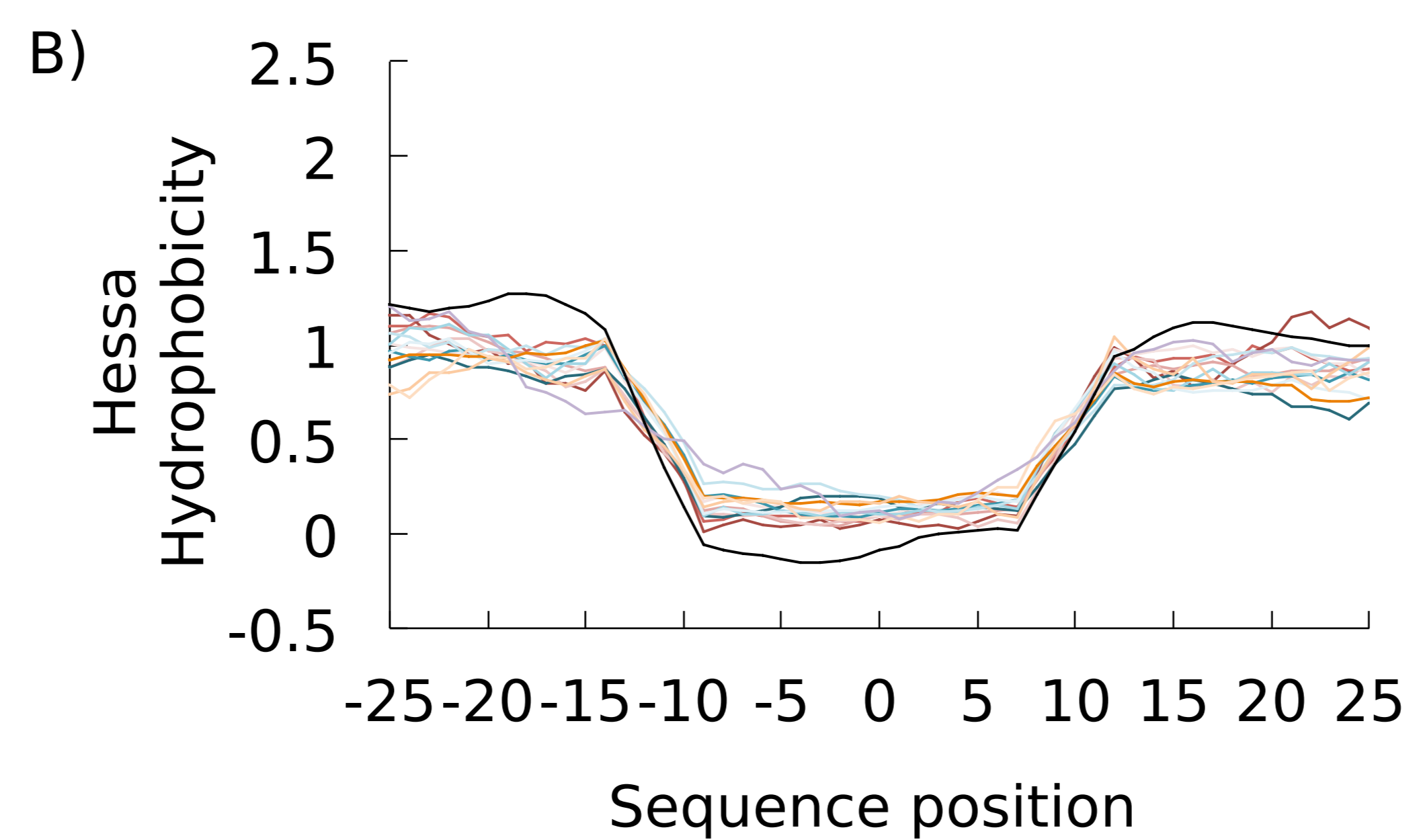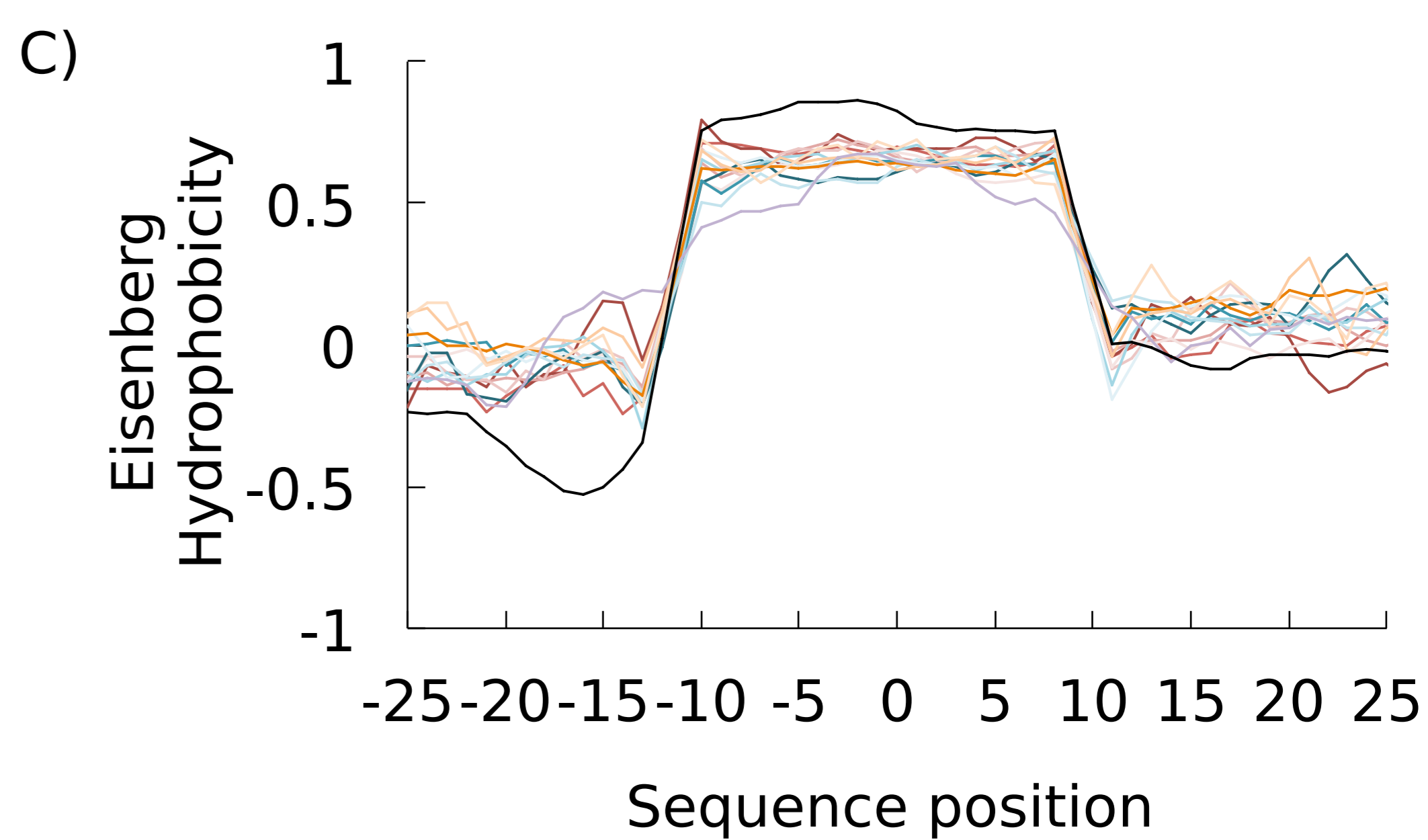

Supplement: Supplementary file 2 — The difference in hydrophobicity between the single-pass and multi-pass datasets stratified by number of TMHs is not due to the choice of scale. As with Fig. 5, UniHuman was stratified according to the number of TMHs in each protein. The mean amino acid hydrophobicity values of TMHs with a sliding unweighted window of 3 residues from UniHuman proteins at each position were plotted. To validate the findings presented in Fig. 5a, several scales of hydrophobicity were used. (A) The White and Wimley whole residue scale [53] is based on the partitioning of peptides between water and octanol as well as water to POPC. A positive score indicates a more polar score. (B) The Hessa biological scale [36]. The hydrophobicity values represent the free energy exchange during recognition of designed peptide TMHs by the endoplasmic reticulum Sec61 translocon and, therefore, negative values indicate an energetic preference for the interior of a lipid bilayer. (C) Eisenberg’s consensus scale [54] is a scale based on the earlier scales from Nozaki and Tanford [86], Wolfenden et al. [87], Chothia [88], Janin [89] and the von Heijne and Blomberg scale [90]. The scales are normalised according to serine. A positive score indicates a generally more hydrophobic score. (PDF 43 kb) [file 12915_2017_404_MOESM2_ESM.pdf]

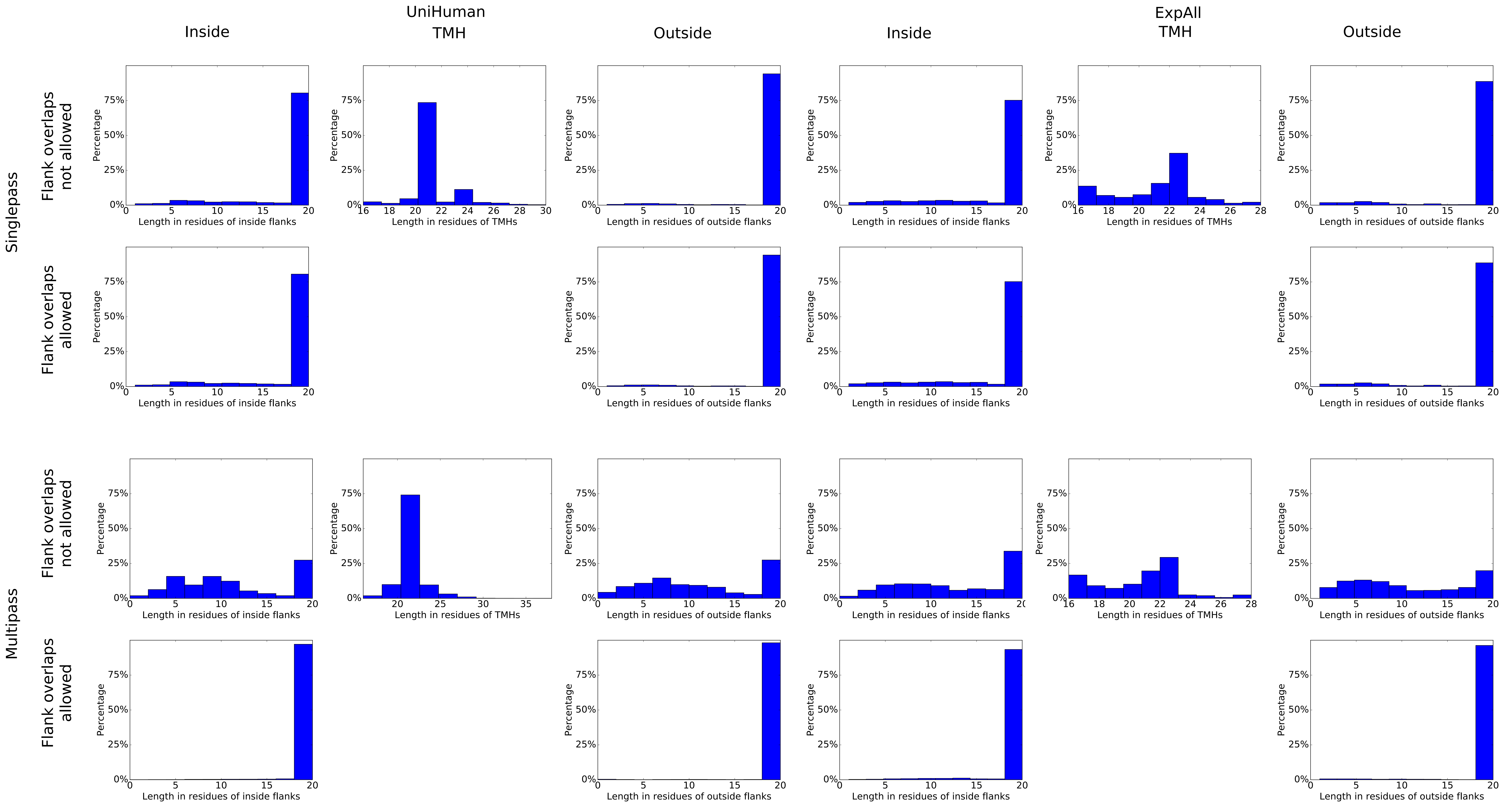

Supplement: Supplementary file 4 — The lengths of flanks and TMHs in multi-pass and single-pass proteins in the UniHuman and ExpAll dataset. On the horizontal axis are the lengths of the TM segment regions in residues. On the vertical axis are the percentages of the population. There are three regions: the inside flank, the TMH and the outside flank. These regions are acquired according to the TMH boundary of the respective database. Where no overlap is permitted, if the flank encroaches the flank of another TMH, the flank length becomes half the number of residues in the loop region between the two features. Where they are allowed to overlap, flanking residues may include other flanks, or indeed other TMHs. (PDF 410 kb) [file 12915_2017_404_MOESM4_ESM.pdf]

A)

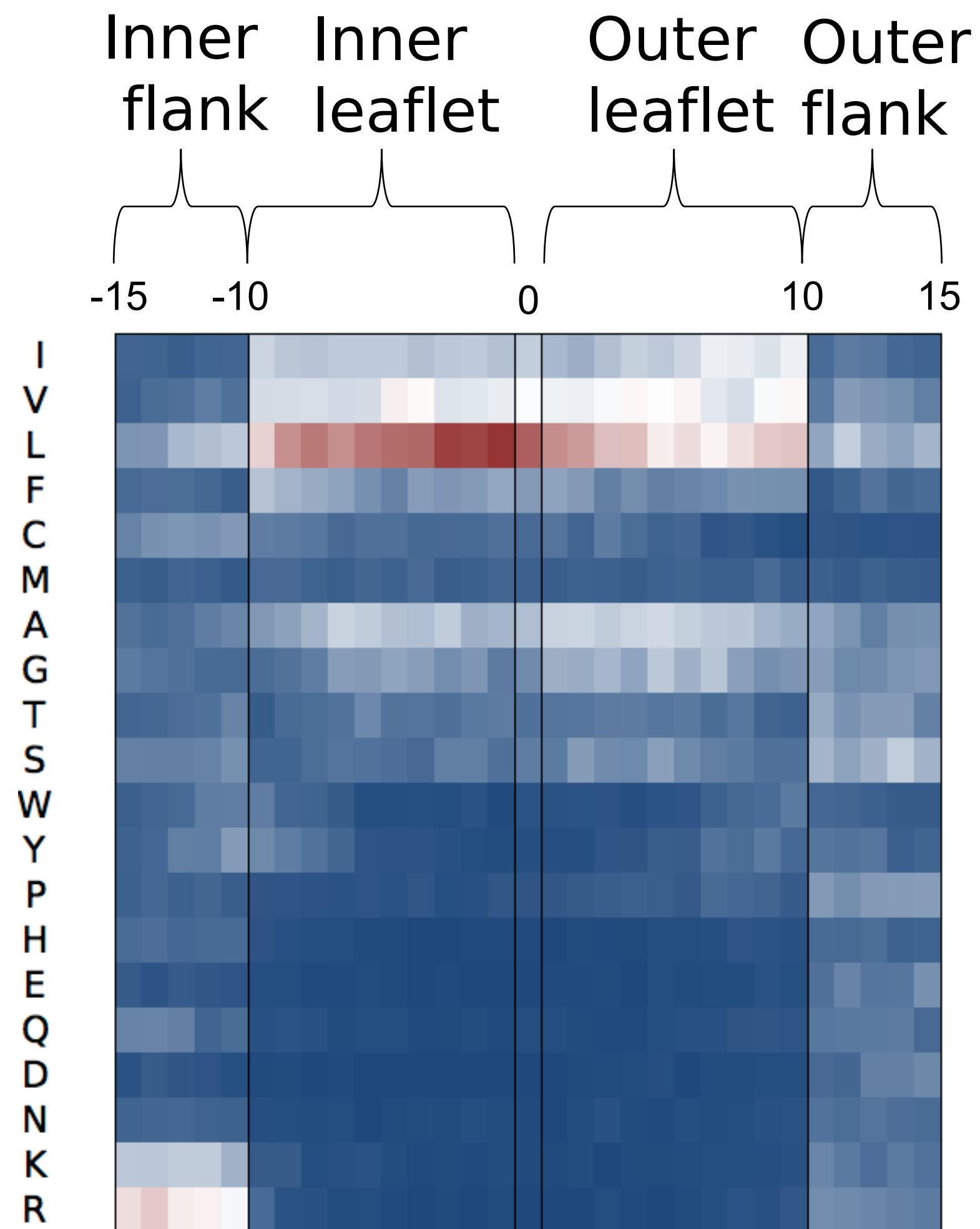

$$p_{i,r} = \frac{a_{i,r}}{\max_r(a_r)}$$

B)

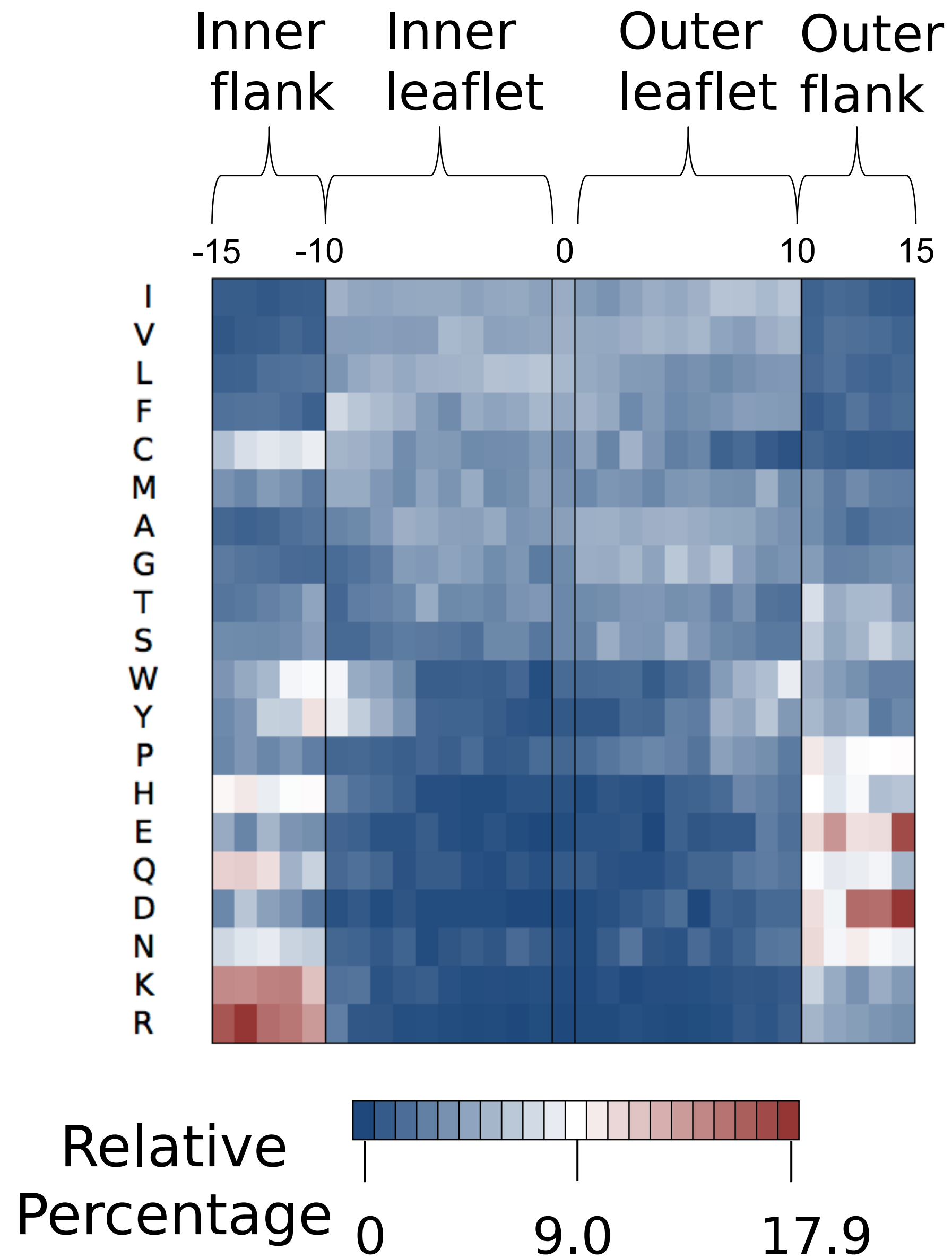

$$q_{i,r} = \frac{100 \cdot a_{i,r}}{a_i}$$

Supplement: Supplementary file 6 — Relative percentage heatmaps from the predictive datasets calculated by fractions of the absolute maximum and by the relative percentage of a given amino acid type. The residue position aligned to the centre of the TMH is on the horizontal axis, and the residue type is on the vertical axis. Amino acid types are listed in order of decreasing hydrophobicity according to the Kyte and Doolittle scale [52]. The flank lengths in the TMH segments were restricted to up to ±5 residues. The scales for each heatmap are shown beneath the respective subfigure. All TMHs and flank lengths are from the UniHuman dataset. (A) The heatmap has been coloured according to a scale that uses column-wise normalisations used in previous studies [9]. See Eq. (1) in the Methods section. As an illustrative example, we show how the value for E at position ±12 is obtained. There are in total 91/22 Es at these positions in 1705 sequences; thus, the represented value is 0.013 at –12 and 0.053 at 12. Note that L is clearly a hotspot as well as trends for other hydrophobic residues, I and V, as is to be expected. A positive inside effect can also be seen. (B) The heatmap has been coloured according to the relative percentage of each amino acid type. Here, 91/22 Es at position ±12 are compared with 615 Es seen within the flanks and the TMH section itself amongst all sequences in the alignment. So, the expectation of an E at position ±12 if there is any E in the TMH + flanks region at all is 0.036 at –12 and 0.148 at position 12. With this type of normalisation, not surprisingly, we see the positive-inside rule is hotter than in subfigure A. There are also hotspots in the flanks for the negatively charged residues on the outside flank. The leucine hotspot is no longer very pronounced, as the leucines are quite evenly spread over many positions. (PDF 120 kb) [file 12915_2017_404_MOESM6_ESM.pdf]
