# Supplementary material for: Charged residues next to transmembrane regions revisited: “Positive-inside rule” is complemented by the “negative inside depletion/outside enrichment rule”
Source: BMC Biol. 2017 Jul 24;15:66. doi: 10.1186/s12915-017-0404-4 (PMC5525207; doi:10.1186/s12915-017-0404-4)
Supplement: Supplementary file 3 — The experimental evidences of TOPDB. The total number of experimental evidences that contribute to ExpAll according to the TOPDB database (more information is available at http://topdb.enzim.hu/?m=exptype&mid=14). * refers to the total number of a subsection being larger than the total of the subcategories, likely due to lack of annotation where ambiguous literature evidence is counted towards the total but cannot be categorised further. (DOC 47 kb) [file 12915_2017_404_MOESM3_ESM.doc]

**Table S1. The experimental evidences of TOPDB.**

The total number of experimental evidences that contribute to ExpAll according to the TOPDB database (More information at http://topdb.enzim.hu/?m=exptype&mid=14). “*” refers to the total number of a subsection being larger than the total of the subcategories, likely due to lack of annotation where ambiguous literature evidence is counted toward the total, but cannot be categorised further.

| **Experiment** | | **Bitopic (Single-pass)** | **Polytopic (Multi-pass)** |
| --- | --- | --- | --- |
| **Fusion** | PhoA | 97 | 2332 |
|  | PhoAS | 0 | 90 |
|  | LacZ | 20 | 433 |
|  | PhoALacZ | 0 | 224 |
|  | BlaM | 162 | 570 |
|  | BAD | 0 | 2 |
|  | PL | 0 | 47 |
|  | GFP | 18 | 591 |
|  | HIS | 4 | 2 |
|  | SplitUbiquitin | 0 | 11 |
|  | Suc2 | 0 | 96 |
|  | Other | 1 | 137 |
|  | Total Fusion | 316* | 4600* |
| **PostTransMod** | NGlyc | 4634 | 1130 |
|  | Cman | 0 | 6 |
|  | Phosphorylation | 4 | 1 |
|  | Ubiquitination | 47 | 102 |
|  | Total PostTransMod | 4685 | 1239 |
| **Protease** | Partial Proteolysis | 51 | 264 |
|  | Signal Peptidase | 1 | 0 |
|  | TID | 13 | 15 |
|  | Total Protease | 64 | 279 |
| **Immunolocalisation** | Epitope Insertion | 33 | 313 |
|  | Endogen Epitope | 8 | 41 |
|  | Total Immunolocalisation | 53* | 451* |
| **Chemical modification** | Cys | 0 | 361 |
|  | Lys | 0 | 3 |
|  | Quenching | 0 | 2 |
|  | Total Chemical Modification | 0 | 368* |
| **Structure** | PDBTM TMDET | 5968 | 41977 |
| **Other** | Revertants | 0 | 14 |
|  | SeqMotif | 2 | 32 |
|  | Tailoring | 1 | 67 |
|  | Total other | 3 | 115* |
