# Supplementary material for: Charged residues next to transmembrane regions revisited: “Positive-inside rule” is complemented by the “negative inside depletion/outside enrichment rule”
Source: BMC Biol. 2017 Jul 24;15:66. doi: 10.1186/s12915-017-0404-4 (PMC5525207; doi:10.1186/s12915-017-0404-4)
Supplement: Supplementary file 5 — Records with INTRAMEM and TRANSMEM flanking region overlap. The total number of TMHs from UniProt datasets with flanking region overlap between INTRAMEM and TRANSMEM regions. The number of multi-pass records to which the TMHs belong are shown in brackets. (DOC 39 kb) [file 12915_2017_404_MOESM5_ESM.doc]

**Table S2. Records with INTRAMEM and TRANSMEM flanking region overlap.**

The total number of TMHs from UniProt datasets with flanking region overlap between INTRAMEM and TRANSMEM regions. The number of multi-pass records that the TMHs belong to are shown in brackets.

| **Dataset** | **Flank length** | | | | | |
| --- | --- | --- | --- | --- | --- | --- |
|  | **5** | | **10** | | **20** | |
|  | **Single-pass** | **Multi-pass** | **Single-pass** | **Multi-pass** | **Single-pass** | **Multi-pass** |
| **UniHuman** | 0 | 96 (80) | 1 | 151 (90) | 5 | 204 (96) |
| **UniER** | 0 | 6 (6) | 1 | 13 (8) | 1 | 16 (8) |
| **UniGolgi** | 0 | 1 (1) | 0 | 2 (2) | 0 | 4 (2) |
| **UniPM** | 0 | 57 (46) | 0 | 93 (51) | 3 | 113 (52) |
| **UniCress** | 0 | 17 (17) | 0 | 24 (18) | 0 | 46 (18) |
| **UniFungi** | 0 | 0 | 0 | 0 | 0 | 0 |
| **UniBacilli** | 0 | 11 (3) | 0 | 12 (3) | 0 | 13 (3) |
| **UniEcoli** | 0 | 22 (8) | 0 | 25 (9) | 0 | 31 (9) |
| **UniArch** | 0 | 0 | 0 | 8 (8) | 0 | 17 (9) |
